# Dynamic modeling in neurocognitive frameworks of childhood ADHD: a review of inhibitory control and reward systems

**DOI:** 10.1038/s41398-026-03972-0

**Published:** 2026-03-23

**Authors:** Weidong Cai, Yoshifumi Mizuno

**Affiliations:** 1 Department of Psychiatry and Behavioral Sciences, Stanford University, Stanford, CA 94305 USA; 2 Wu Tsai Neurosciences Institute, Stanford University, Stanford, CA 94305 USA; 3 Wu Tsai Human Performance Alliance, Stanford University, Stanford, CA 94305 USA; 4 Maternal & Child Health Research Institute, Stanford University, Stanford, CA 94305 USA; 5 Stanford Institute for Human-Centered Artificial Intelligence, Stanford University, Stanford, CA 94305 USA; 6 Research Center for Child Mental Development, University of Fukui, Fukui, 910-1193 Japan; 7 Division of Developmental Higher Brain Functions, University of Fukui, Fukui, 910-1193 Japan; 8 Department of Child and Adolescent Psychological Medicine, University of Fukui Hospital, Fukui, 910-1193 Japan

**Keywords:** Neuroscience, ADHD

## Abstract

Cognitive models of attention deficit hyperactivity disorder (ADHD) have traditionally centered on deficits in inhibitory control and motivation. A substantial body of empirical research supports impairments in both domains: children with ADHD exhibit elevated commission error rates, prolonged stop-signal reaction time, and a strong preference for smaller immediate rewards over larger delayed ones. In the first part of this review, we synthesize key findings from behavioral and task-state functional neuroimaging studies that characterize these deficits, highlighting how deficits in inhibitory control and motivation/reward systems manifest across tasks and contexts. We further examine evidence on the interplay between the two systems and how their interaction is altered in children with ADHD. In the second part, we introduce recent advances in cognitive and computational neuroscience that extend classic neurocognitive models by incorporating dynamic perspectives. Specifically, we describe the dynamic dual-control framework, which distinguishes between proactive and reactive inhibitory control, and highlight dynamics of trial-by-trial adaptive response strategy adjustment. We also review emerging computational models that can identify latent brain states and their evolution during task performance, revealing novel neurodynamic mechanisms that may underlie cognitive instability in ADHD. Additionally, we discuss pharmacological studies that shed light on how the inhibitory control and motivation/reward systems are modulated by medication. Finally, we address current limitations and challenges in ADHD research, with a particular focus on task-state functional neuroimaging, and propose future directions for advancing the field. By integrating dynamic modeling approaches with established neurocognitive frameworks, this review aims to deepen our understanding of the brain mechanisms underlying ADHD and to inform the development of more precise and effective interventions.

Attention deficit hyperactivity disorder (ADHD) is a neurodevelopmental disorder with a significant global prevalence, estimated between 5–10% in the pediatric population [1–5]. In the United States, the diagnosis of childhood ADHD has risen markedly over the past three decades [6, 7]. According to the 2022 National Survey of Children’s Health, 11.4% of U.S. children aged 3–17 years had ever received an ADHD diagnosis, with 10.5% meeting criteria for current diagnosis based on parents’ report that the child has been diagnosed [8]. This substantial increase has spurred concerns and ongoing debate regarding the underlying pathophysiology of ADHD and the etiological factors contributing to these diagnostic trends [9].

Early diagnosis of childhood ADHD most commonly occurs between ages 5 and 7, when persistent and developmentally inappropriate behaviors, such as difficulty in remaining seated, frequent task interruptions, and consistent failure to follow instructions, become increasingly apparent and disruptive in structured settings like school. These behaviors are not isolated incidents but rather frequent and pronounced manifestations of the disorder’s core symptoms: inattention and hyperactivity/impulsivity. They often lead to academic underachievement, challenges in peer relationships, and increased family stress, which commonly prompt caregivers or educators to seek professional evaluation. Early identification is therefore critical, as it enables timely interventions that can improve long-term functional outcomes [10, 11].

Theoretical models of ADHD have long proposed that behavioral symptoms arise from underlying deficits in inhibitory control and motivation systems [12–21], both of which are essential for goal-directed behaviors [22, 23]. Empirical research has shown that children with ADHD tend to perform worse than typically developing (TD) peers on tasks involving inhibitory control and reward-related decision making [24, 25]. Findings from functional neuroimaging studies further reveal atypical activity in brain networks related to these cognitive functions during both rest and task engagement [26, 27]. However, despite these promising results, weak and inconsistent effect sizes have been reported [28, 29], casting doubt on the explanatory power of these neurocognitive models, thus limiting their clinical utility. From a clinical standpoint, these neurocognitive models have yet to yield reliable biomarkers that can be used for diagnostic, prognostic, or treatment monitoring purposes.

Human cognition relies on the brain’s capacity to flexibly and effectively allocate cognitive resources in response to moment-to-moment changes in task demands [30–36]. Understanding the underlying brain dynamics, encompassing transient and time-varying interactions among distributed brain areas, during task performance is thus essential for developing a comprehensive neurocognitive framework to investigate cognitive deficits and behavioral dysfunctions. Early neuroscience models of ADHD posited that unstable brain states may underlie attentional difficulties in affected children [37]. However, most of the existing research has focused on average neural responses, overlooking critical features of functional brain dynamics that contribute to clinical symptoms of ADHD. Integrating dynamic perspectives into clinical research offers the potential to uncover previously unrecognized neurocognitive mechanisms that drive behavioral problems associated with the disorder.

In this review, we examine behavioral and neuroimaging evidence supporting inhibitory control and motivation/reward models of ADHD. Our focus is specifically on task-state fMRI findings, as they capture brain responses to cognitive demands. While resting-state fMRI has generated a large body of research, it lacks the ability to probe how the brain dynamically responds to goal-directed tasks. We also highlight emerging research that integrates neurodynamic frameworks into these models, aiming to provide a more comprehensive and mechanistic understanding of the disorder’s neurobiological underpinnings.

## Inhibitory control and childhood ADHD

Inhibitory control is a fundamental function that regulates an individual’s actions, emotions, and thoughts. This ability develops progressively throughout childhood and adolescence, reaching maturity in early adulthood [38, 39]. Notably, middle childhood (8–10 years old) represents a particularly critical period for the development of inhibitory control [40] and coincides with the typical diagnosis of childhood ADHD [41]. According to Barkley’s inhibitory control model of ADHD, many behavioral issues observed in children with the disorder stem from difficulties in suppressing urges and impulses that are inappropriate or no longer relevant for the context [12].

**Fig. 1:**
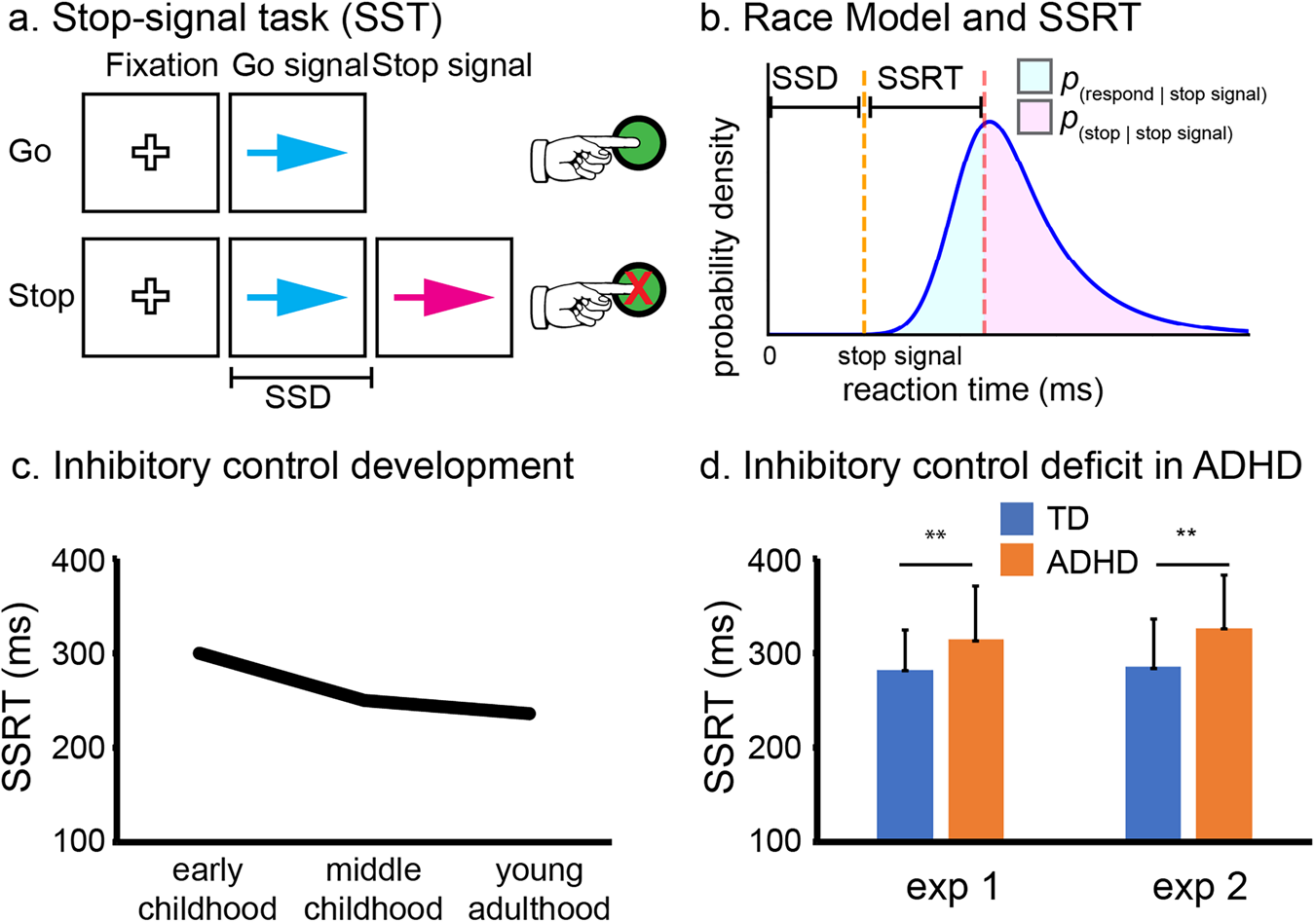
Inhibitory control paradigm, model, development and deficits in ADHD. **a** Stop-signal task (SST) involves frequent Go trials and infrequent Stop trials. Participants are instructed to make accurate and speedy responses (e.g. button press) to go signals (e.g. a blue arrow) and withhold their response tendency when a go signal is shortly followed by a stop signal (e.g. the blue arrow turns to red). The critical parameter in the SST is the stop-signal delay (SSD), the interval between the onsets of go and stop signals, whose values are typically adjusted trial-by-trial with a performance-based staircase procedure. **b** The Race model can estimate stop-signal reaction time (SSRT), which quantifies how fast one can cancel a prepotent response [46]. **c** SSRTs become shorter through childhood, indicating development of inhibitory control functions. The plot illustrates averaged SSRTs from early and middle childhood to young adulthood. Data were obtained from the two previous studies [38, 39]. **d** Children with ADHD have elongated SSRTs in comparison to TD children, suggesting inhibitory control deficits in ADHD. The plot is adapted from a previous study in which two experiments (exp1 and exp2) show similar inhibitory control deficits in children with ADHD (9–12 years old) [50].

Inhibitory control is hypothesized to be a domain-general, cross-modality process, with similar mechanisms underlying inhibition of both motor and cognitive processes [42–45]. Because of the practical advantage in validation and measurement, motor response inhibition is most commonly studied in an experimental setting. Various task paradigms, such as Go/NoGo task (GNGT), Stop-signal task (SST), Flanker task, Stroop task, and anti-saccade task have been developed to examine inhibitory control over actions. These tasks typically include two trial types: low-demand and high-demand of inhibitory control. The high-demand trials require participants to cancel or override prepotent, reflexive, or automatic responses that are inappropriate in a given context, such as in no-go, stop, incongruent, and anti-saccade conditions (Fig. 1a). Among these paradigms, the SST is unique as it allows estimation of stop-signal reaction time (SSRT) via the Race Model, providing a quantitative measure of how quickly an individual can inhibit dominant response tendency [46, 47] (Fig. 1b). Notably, neurophysiological evidence from non-human primate studies has demonstrated strong alignment between SSRTs and the timing of neuronal activity associated with action cancellation, supporting the neurobiological validity of SSRT [48, 49].

The SSRT improves steadily from childhood through adolescence, typically reaching a plateau in early adulthood [38, 39] (Fig. 1c). Numerous studies have shown that children with ADHD exhibit longer SSRTs compared to their typically developing (TD) peers (Fig. 1d) and slower SSRTs are associated with more severe ADHD symptoms [50–52]. Meta-analytic research confirms that SSRT yields a median effect size (~0.6) in distinguishing children with ADHD versus TD children, surpassing the effect size of commission errors on the GNG task (~0.5), another widely used behavioral index of inhibitory control [25]. These findings suggest that children with ADHD have relatively weaker inhibitory control over actions, aligning with the inhibitory control model of the disorder [12].

## Dynamic dual control model and ADHD

**Fig. 2:**
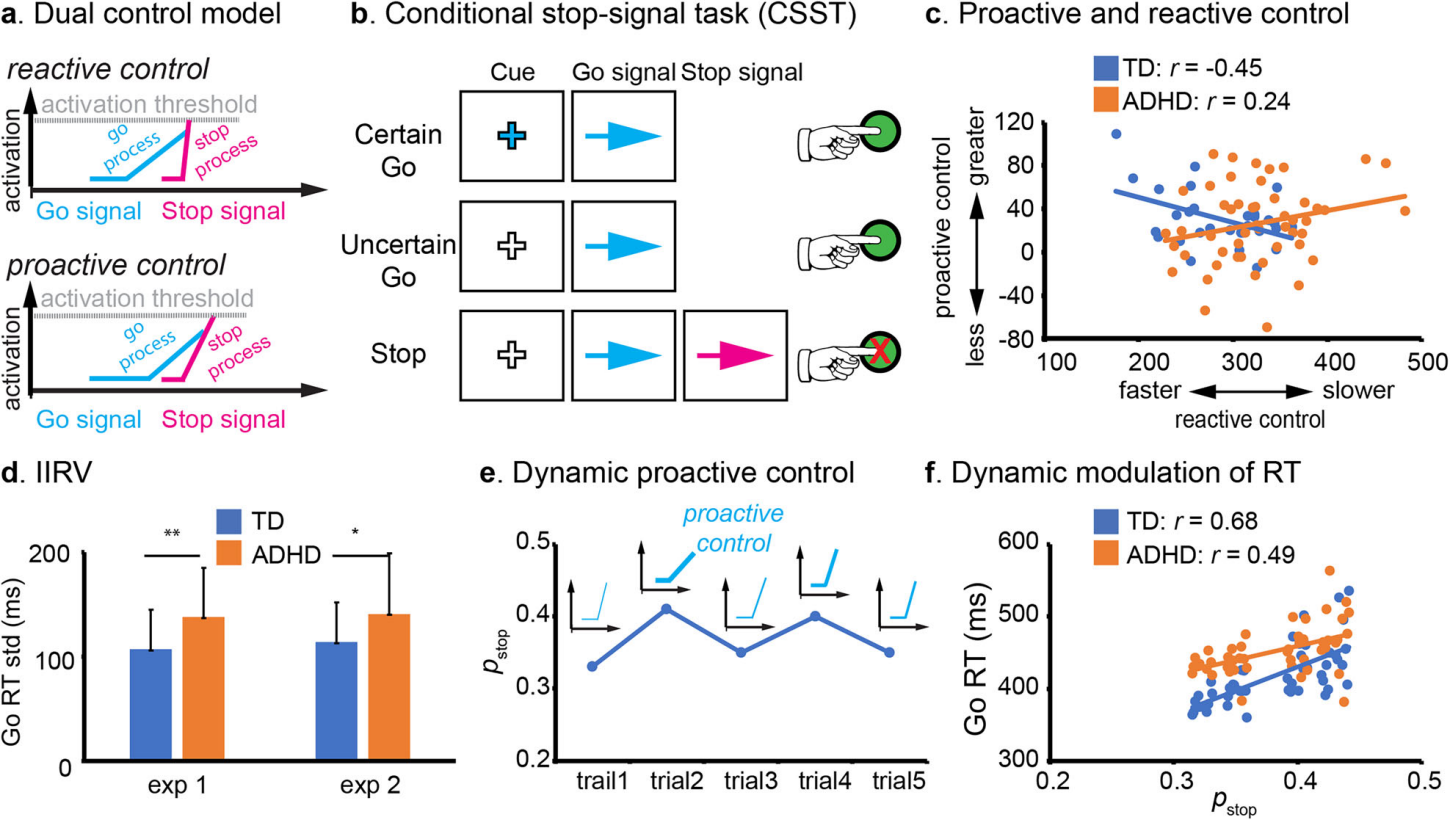
Dynamic dual inhibitory control model. **a** The dual inhibitory control model distinguishes between reactive and proactive control processes. Reactive control involves the suppression of a dominant motor, cognitive, or emotional response triggered by the detection of conflict or interference, whereas proactive control involves the anticipatory deployment of inhibitory mechanisms based on prior knowledge or contextual cues that signal a high likelihood of upcoming conflict. In the stop-signal task, reactive control corresponds to the stop process initiated by the infrequent stop signal, aiming to reach its activation threshold before the go process completes; whereas proactive control is reflected in strategically slowing of the go process, delaying its progress toward the activation threshold in anticipation of a potential stop signal. **b** The conditional stop-signal task (CSST) is an experimental paradigm designed to probe dual mechanisms of inhibitory control. In this task, the Certain Go cue (blue cross) signals a go trial with no probability of a stop signal, thus involving no inhibitory control demand. In contrast, the Uncertain Go cue (white cross) introduces the possibility of a stop signal appearing shortly after the go signal, thereby engaging proactive inhibitory control mechanisms due to increased demand for response inhibition. Proactive control is typically measured by increased reaction time (RT) in the Uncertain Go than Certain Go conditions. **c** A previous study has revealed that greater proactive control is associated with faster reactive control functions in TD children (*r* = −0.45) but not in children with ADHD [50]. Reactive control is quantified using the SSRT. Proactive control is calculated as the difference in RT between Uncertain Go and Certain Go trials. **d** Children with ADHD have higher intra-individual response variability (IIRV) than TD children. The plot is adapted from a previous study in which two experiments (exp1 and exp2) show heightened IIRV in children with ADHD (9–12 years old) [50]. **e** Based on trial history, participants continuously update their belief about the probability of stop signal (p_stop_) in the coming trial and adjust response strategies (proactive control) accordingly. **f** A previous study has shown that trial-wise anticipation of stop signal (p_stop_) is strongly associated with RT in TD children and such modulation is weaker in children with ADHD [50]. Two exemplar participants’ data are illustrated (1 TD and 1 ADHD).

Despite the long-standing view that inhibitory control is a single cognitive process, more recent dual control models propose that inhibitory control operates through two distinct modes: reactive and proactive control [53, 54] (Fig. 2a). Reactive inhibitory control refers to the suppression of a prepotent motor, cognitive, or emotion response upon detecting conflict or interference. In a classroom setting, this is exemplified by a child who impulsively starts to shout out an answer but manages to stop immediately upon realizing they did not raise their hand. In SST, reactive control is typically quantified using the SSRT. Calculated via the Race Model, the SSRT provides an estimate of the latency of the unobservable inhibitory process, representing the speed at which an individual can reactively halt a response once a stop signal is presented.

In contrast, proactive inhibitory control involves the anticipatory deployment of inhibitory mechanisms based on prior knowledge or contextual cues that signal the likelihood of upcoming conflict. For instance, a student acts proactively when they consciously slow down their movements and quiet their voice before entering a library, anticipating the requirement for silence. Although both processes contribute to performance on inhibitory control tasks, most behavioral measures, such as commission errors, stop accuracy, and SSRT, are primarily designed to assess reactive inhibitory control. Proactive inhibitory control, on the other hand, is examined by manipulating task demand, such as varying the probability of inhibitory signals (e.g. stop or no-go signals) (Fig. 2b). Operationally, proactive control is calculated as the magnitude of response slowing, specifically, the strategic prolongation of reaction time on Go trials in contexts with a high probability of stop signals compared to low probability contexts. A great degree of response slowing indicates a robust engagement of proactive strategies to prevent commission errors.

Support of the dual control model comes from multiple lines of evidence: (1) correlation between SSRT and response slowing [55], (2) similar suppression effects on motor excitability in both control modes [56, 57], and (3) overlapping neural substrates underlying reactive and proactive inhibitory control processes [58]. Like reactive inhibitory control, proactive inhibitory control is under development during childhood [59–62].

Although numerous studies have examined reactive inhibitory control in childhood ADHD, research on proactive inhibitory control remains limited and findings are mixed [50, 63–65]. For example, Van Hulst et al. used a modified SST incorporating anticipatory cues to examine reactive and proactive inhibitory control in TD children and those with ADHD symptoms and other neurodevelopmental disorder symptoms [65]. The cues indicated varying probability of stop signals, and proactive inhibitory control was assessed via the interaction between stop signal probability and reaction time on go trials. Although they found a robust proactive control effect, no significant between-group differences were observed. In another study that manipulated stop signal probabilities within the classic SST framework, researchers reported that children with ADHD exhibited similar levels of response slowing as TD children when provided with explicit task cues [50]. However, when proactive control strategies had to be learned implicitly through experience, by tracking trial history and action consequences, children with ADHD were less efficient in utilizing such strategies [50]. Interestingly, TD children with shorter SSRTs also showed greater response slowing, suggesting a coordinated interaction between proactive and reactive inhibitory control mechanisms, similar to patterns seen in adults [50, 55]. This coordinated engagement was absent in children with ADHD [50] (Fig. 2c), implying not only deficits in reactive control, but also challenges in integrating and coordinating both inhibitory control modes even though proactive inhibitory control can be implemented under explicit instruction.

Inhibitory control tasks often engage implicit learning processes, as local probability of task-relevant signals (e.g., no-go or stop signals) varies from trial to trial, even though the overall task structure remains constant. As a result, dynamically updating expectation based on recent trial history is critical for accurate anticipation, strategic adjustment, and optimal behavioral performance. Traditional behavioral metrics, such as mean accuracy and reaction time (RT), are static measures that fail to capture trial-by-trial variability in cognitive processing. Measures of intra-individual response variability (IIRV), such as the standard deviation, sigma, or tau of RT [66, 67], have been used to quantify behavioral fluctuation during task performance. IIRV is often used as an index of attentional lapses, and studies consistently show that children with ADHD exhibit greater IIRV than TD children, reflecting less consistent task engagement and potentially poorer sustained attention [25, 68–71] (Fig. 2d). However, IIRV has notable limitations. First, it is a coarse metric that aggregates data across trials and lacks the temporal resolution to capture dynamic processes as they evolve over time. Second, variability in RT can stem from multiple sources, such as arousal regulation, fatigue, attentional lapses, or strategic adjustment [71–73], but IIRV cannot disentangle these contributing factors.

To better understand the dynamic cognitive mechanisms underlying inhibitory control, computational modeling approaches have been employed to estimate trial-by-trial latent processes driving behavioral variability [74, 75]. One such model is the Dynamic Belief Model (DBM), which estimates a subject’s belief about the chance of an upcoming inhibitory signal (p_stop_) based on trial history [75]. According to this model, individuals are expected to exert greater proactive control when they believe the probability of an inhibitory signal is increasing (Fig. 2e). This prediction has been supported by strong positive correlations between trial-wise p_stop_ and RT in adult participants [75–77], indicating strategic modulation of behavior in anticipation of inhibitory demands. A similar association has been observed in children, suggesting that they are capable of making strategic, history-based adjustment in proactive control. However, children with ADHD exhibit weaker correlation between trial-wise p_stop_ and RT compared to their TD peers [50], indicating a reduced capacity to update belief based on trial history and/or adjust proactive control strategies accordingly (Fig. 2f).

The dynamic dual control model distinguishes between proactive and reactive inhibitory control mechanisms, characterizing their continuous adaptation to changing task demands. Integrating this framework into models of inhibitory control offers a more comprehensive understanding of cognitive deficits in children with ADHD by capturing the temporal variability, multifaceted cognitive processes, and strategic adjustment that underlie inhibitory control functions.

## Neural mechanism of inhibitory control and ADHD

**Fig. 3:**
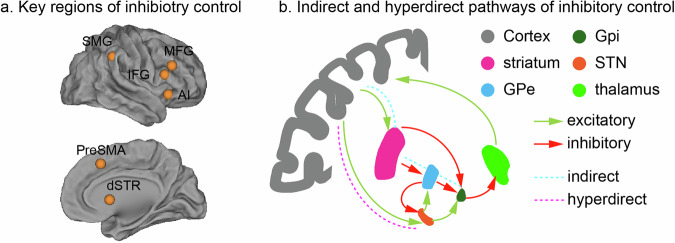
Cortical-subcortical regions involved in inhibitory control. **a** Inhibitory control tasks, such as stop-signal task and go/no-go task, commonly recruit the anterior insula (AI), inferior frontal gyrus (IFG), middle frontal gyrus (MFG), pre-supplementary motor area (preSMA), supramarginal gyrus (SMG) and dorsal striatum (dSTR), with activation dominance in the right hemisphere. The coordinates of these plotted regions were obtained from meta-analysis of 70 functional neuroimaging studies in which the stop-signal task and go/no-go task were used to examine brain activation during inhibitory control in adult participants [78]. **b** Indirect and hyper-direct pathways of cortical-basal ganglia circuits have been implicated in inhibitory control of actions.

Inhibitory control engages a widely distributed network of cortical and subcortical regions. Meta-analytic research has identified consistent activation in key areas, including the anterior insula, inferior frontal gyrus, middle frontal gyrus, pre-supplementary motor cortex, supramarginal gyrus and dorsal striatum, with a dominant involvement of the right hemisphere during response inhibition [40, 78, 79] (Fig. 3a). In particular, the indirect and hyper-direct pathways of basal ganglia have been implicated in inhibitory control of motor responses [80, 81] (Fig. 3b). It has been suggested that the subthalamic nucleus, a critical node of the hyper-direct pathway, plays a vital role in rapid inhibition [82]. Although the subthalamic nucleus is not frequently reported in task-state neuroimaging studies of inhibitory control, its importance has been substantiated by both animal studies and human clinical research [40, 83–86]. Task-dependent connectivity analyses further demonstrate that inhibitory control modulates interactions both within canonical cortical networks and between cortical and subcortical regions [40, 84, 87–91], highlighting the system-wide communication necessary for effective inhibitory control. However, few studies have examined task-related activation and connectivity of the subthalamic nucleus in children with ADHD.

Inhibitory control paradigms, such as SST and GNGT, have been widely used to probe abnormal brain activation in ADHD. Numerous studies have reported hypoactivation in core inhibitory control regions, including inferior frontal gyrus and dorsomedial prefrontal cortex, in children and adolescents with ADHD compared to TD peers [51, 92–106]. However, findings are mixed, with some studies reporting no significant between-group differences [107–109]. Notably, many earlier neuroimaging studies had small sample sizes (*n* < 20 per group), limiting their generalizability. A larger study involving 74 adolescents with ADHD and 74 controls found hypo-activation in lateral and medial prefrontal cortex and striatum during a Go/No-Go task [102]. When making commission errors (i.e. failed inhibition), adolescents with ADHD also showed reduced activation in the anterior insula and anterior cingulate cortex, core nodes of the salience/error detection system, and failed to downregulate default mode network, unlike the controls. These results, replicated in other studies [107, 110], suggest that children with ADHD are less likely to engage salience/error detection system in response to errors. Meta-analytic research further confirmed consistent hypoactivation of the salience and frontoparietal networks across cognitive tasks requiring motor inhibition in individuals with ADHD [26, 111], linking the disorder to impaired recruitment of inhibitory control networks.

Fewer studies have investigated how inhibitory control modulates functional connectivity in children with ADHD [107, 112]. Using generalized psychophysiological interaction analysis [113], Cai et al. examined task-dependent connectivity during a Go/NoGo task in 46 children and adolescents with ADHD and 51 TD children and adolescents acquired in the International Study to Predict Optimized Treatment in ADHD trial [107]. They found that connectivity between dorsolateral prefrontal cortex and posterior parietal cortex, two key nodes in the frontoparietal network, was significantly weaker in children with ADHD than in TD children during No-Go trials. Similarly, van Rooji et al. reported reduced connectivity between inferior frontal gyrus and basal ganglia, as well as between superior frontal gyrus and thalamus and operculum, during successful stop trials in children with ADHD [112]. These findings highlight altered cortical-cortical and cortical-subcortical connectivity patterns in response to inhibitory control demand in children with ADHD, although further research is needed to confirm their reproducibility.

Overall, early task-state functional neuroimaging studies generally agree that children with ADHD show deficits in upregulating inhibitory control system and/or downregulating default mode system in response to increased demand of inhibitory control.

## Motivation, reward-related processes and ADHD

Despite its popularity, the inhibitory control theory has faced considerable scrutiny within the field. First, deficits in inhibitory control are not unique to ADHD; they are also observed in other neurodevelopmental disorders, such as autism [114] and learning disorder [115]. Second, several studies have reported comparable performance on inhibitory control tasks between individuals with and without ADHD, raising questions about the universality of such deficits among those diagnosed. Third, some behavioral difficulties commonly attributed to ADHD may be better explained by impairments in other cognitive domains. As a result, the inhibitory control hypothesis, at least when considered in isolation, does not fully account for the diverse range of behavioral problems observed in children with ADHD.

An alternative and influential theory suggests that many behavioral challenges in ADHD may stem from reduced motivation, especially in tasks requiring sustained cognitive or physical efforts [116–118]. Some researchers have proposed that impairments in inhibitory control may, in fact, be secondary to more fundamental deficits in motivation [17, 119, 120]. This view is supported by a growing body of evidence. Studies using self- or teacher-reported questionnaires and observation methods have found that children with ADHD tend to be less motivated to engage in academic tasks compared to their TD peers [121–123], although findings are somewhat mixed [124]. For example, one recent study found that children and adolescents with ADHD reported particularly low motivation for cognitively demanding tasks [125]. Another study involving word puzzles found that children with ADHD were more likely to abandon the task than TD children, indicating reduced persistence and motivation in academic context [126]. Furthermore, the severity of core ADHD symptoms, such as inattention and hyperactivity, has been linked to diminished academic motivation among kindergarten students [127].

Motivation to complete a task, however, is influenced by a range of factors, including perceived task difficulty relative to one’s abilities and the individual’s self-belief or confidence. It is plausible that children with ADHD may develop low self-efficacy due to repeated academic failure, which in turn reduces their motivation to engage in learning activities [128]. Although experimental research examining motivational processes in non-academic contexts remains limited, several studies offer valuable insights. For instance, one investigation found that adults with ADHD were less willing than controls to engage in repeated manual effort tasks [129]. In another experiment requiring participants to choose between varying levels of cognitive or physical effort, individuals with ADHD showed a general reluctance to invest effort in both types of tasks [130]. However, findings are not entirely consistent: one study reported that children with ADHD were just as likely as TD peers to select high-effort tasks, although their engagement was less consistent [131]. Another study found no significant group differences in physical effort-related discounting [132].

Importantly, both extrinsic and intrinsic motivation, central to human behavior, are strongly influenced by external factors, such as reward [133–135]. Motivation is closely tied to reward sensitivity, and the motivational deficit theory posits that children with ADHD may exhibit altered sensitivity to rewards [119], a feature broadly implicated across various mental health conditions [136]. Numerous studies have examined whether children with ADHD respond differently to rewards compared to their TD peers. One of the most widely used paradigms in this area is the delay discounting task (DDT), which assesses impulsive decision-making and reward sensitivity. In the classical DDT, participants choose between smaller, immediate rewards and larger, delayed ones. Children with ADHD tend to prefer smaller, immediate rewards [137–139], indicating a steeper discounting of delayed gratification. Meta-analyses of DDT studies have reported moderate effect sizes (approximately 0.4–0.5) in distinguishing individuals with ADHD from controls [24, 140, 141].

Taken together, these behavioral findings suggest that children with ADHD exhibit reduced motivation and altered reward sensitivity, particularly characterized by a heightened preference for immediate rewards and difficulty with delayed gratification, relative to their TD peers.

## Neural mechanism of reward-related processes and ADHD

**Fig. 4:**
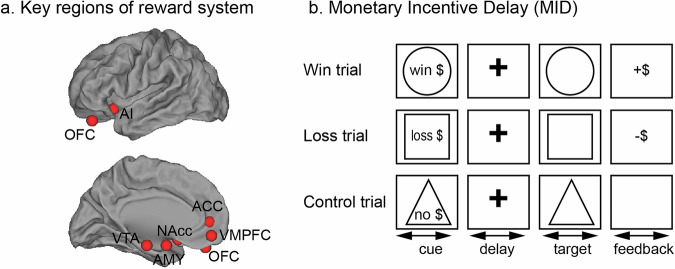
Reward system and task paradigm. **a** Reward processing tasks commonly recruit the ventromedial prefrontal cortex (VMPFC), orbital frontal cortex (OFC), anterior insula (AI), anterior cingulate cortex (ACC), nucleus accumbens (NAcc), amygdala (AMY) and ventral tegmental area (VTA). Coordinates of these regions of interests are obtained from previous meta-analyses of reward-related task fMRI studies [146, 147]. **b** The monetary incentive delay (MID) task is widely used to examine neural substrates underlying reward anticipation and reward outcome [145]. The variable delays between cues and targets allow dissociation between reward anticipation and reward outcome processes.

A broad network of cortical and subcortical regions, including the ventromedial prefrontal cortex, orbital frontal cortex, anterior insula, anterior cingulate cortex, nucleus accumbens, amygdala, and ventral tegmental area, play central roles in motivational and reward-related processes [142–147] (Fig. 4a). One of the most widely used experimental paradigms to investigate these processes in neuroimaging research is the monetary incentive delay task (MID) [145] (Fig. 4b). In this task, participants are presented with cues that signal the potential to win money, avoid losing money, or receive no incentive, followed by a target that requires a rapid response. To maintain a pre-defined success rate, the task difficulty is adaptively adjusted, typically by changing the response time thresholds, yielding different reward outcomes such as win, no-win, loss, and no-loss. The delay period between cue and stimulus presentations is critical in functional neuroimaging studies because it enables the dissociation of brain signals associated with reward anticipation from those related to reward outcome. Meta-analyses of MID studies have consistently found that the nucleus accumbens, anterior insula, and amygdala are strongly activated during reward anticipation, whereas ventromedial prefrontal cortex and orbital frontal cortex are more engaged during reward outcome [148, 149]. In a large-scale adolescent neuroimaging dataset (*n* = 1510) [150], Cao and colleagues revealed widespread activation during reward anticipation including the medial prefrontal cortex, insula, dorsal and ventral striatum, thalamus, and ventral tegmental area. In contrast, activation during reward outcome was more limited, primarily involving the ventromedial prefrontal cortex. Moreover, using psychophysiological interaction methods, the study demonstrated that reward increases functional connectivity between ventral striatum and cortical regions such as the dorsomedial prefrontal cortex, anterior cingulate cortex, and anterior insula during reward anticipation, highlighting the extensive communication between reward and salience networks during reward processing.

The MID has also been extensively employed to examine ADHD-related functional abnormalities in the brain’s reward circuitry [102, 151–161]. Although behavioral differences between ADHD and controls are often minimal in the MID task, a consistent neuroimaging finding is reduced activation in ventral striatum during reward anticipation in individuals with ADHD [102, 151–161]. This hypoactivation is thought to reflect deficits in reward anticipation mechanisms. Additionally, reduced ventral striatum activation during reward anticipation has been linked to hyperactivity in both the MID [151, 157, 159] and DDT [162] tasks, although some studies have reported weak or even opposite effects [156, 161]. Results related to reward outcome have been even more variable. For example, Strohle et al. observed increased activation in right orbitofrontal cortex, left middle frontal gyrus, and inferior frontal gyrus in adults with ADHD compared to controls [159]; Plichta et al. reported greater striatal activation in males with ADHD than controls in response to success outcome [162]; and Stoy et al. found reduced activation in bilateral insula during outcome of loss avoidance [158]. Despite these findings, little is known about task-dependent connectivity during reward-related processes in ADHD.

Together, functional neuroimaging studies provide converging evidence that individuals with ADHD exhibit deficits in engaging key components of the brain’s reward circuit, particularly the ventral striatum, during reward-related processes. These findings underscore the importance of motivational and reward-related functional abnormality in the neurobiology of ADHD.

## Interaction between cognitive control and reward systems

Crucially, inhibitory control and motivation are interrelated processes, rather than isolated functions [22, 163, 164]. The precise mechanism through which changes in motivational states and reward influence inhibitory control are not fully understood. Behavioral research has shown that reward significantly influences individuals’ performance on inhibitory control tasks [165, 166]. Generally, when reward contingencies emphasize response inhibition, individuals tend to adjust their response strategies, leading to greater response slowing (indicative of proactive control) and faster stopping speed (reflecting reactive control) [165, 166]. A substantial body of research has examined the interplay between motivation and inhibitory control in children with ADHD [157, 167–189]. Most of these studies employ classic inhibitory control paradigms such as GNG or SST, incorporating reward manipulation (e.g. varying reward magnitudes or contrasting reward versus loss conditions). Although significant reward effects and group differences in inhibitory control measures are commonly observed, findings regarding the interaction between reward manipulation and ADHD diagnosis are inconsistent [169–190]. The majority of studies report no significant interaction, but a few have found that reinforcement enhances inhibitory control function more in children with ADHD than TD or clinical control groups [171, 177, 182, 185, 187].

Using a reward-modulated inhibitory control paradigm, Boehler et al. found increased activation in the ventral striatum, anterior insula, and anterior cingulate cortex in reward-related than reward-unrelated stop trials, along with enhanced functional connectivity between anterior cingulate cortex and dorsomedial prefrontal cortex [191]. Other studies have similarly shown that reward modulation of inhibitory control engages not only key regions of the reward circuitry but also areas involved in inhibitory control [192–194], a finding supported by meta-analytic research [23]. Despite these insights, few studies have examined the neural mechanisms of reward-modulated inhibitory control in ADHD. Using a Go/NoGo task with low- and high-incentive conditions, Liddle et al. found attenuated deactivation of the default mode network in children with ADHD relative to TD children under low incentive conditions, a difference that disappeared under high incentive conditions [178].

Whereas there is a general agreement that reward enhances inhibitory control, even for children with ADHD, it remains unclear whether this enhancement is greater, less, or equivalent in magnitude compared to children without ADHD. To better understand this variability, it is important to investigate the underlying neural mechanisms of reward-modulated inhibitory control, particularly the dynamic aspects of these mechanisms, which may offer critical insights into individual differences in response to rewards among children with ADHD.

## Neurodynamic models of cognition and ADHD

**Fig. 5:**
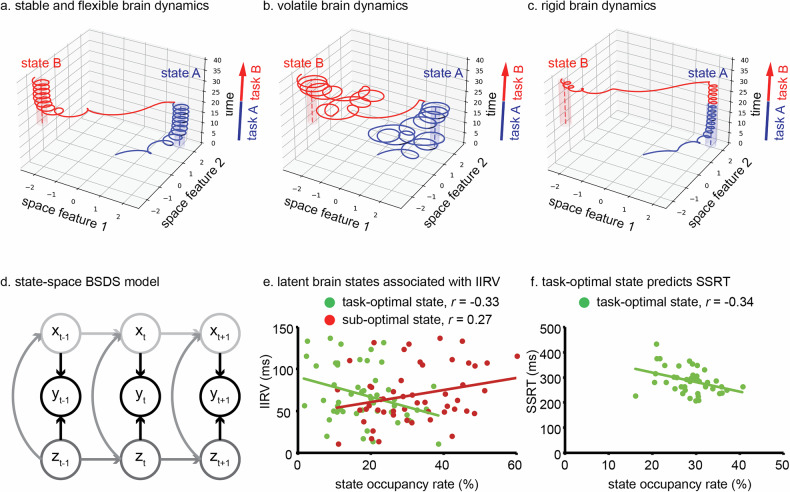
Schematic illustration of stable and flexible, volatile and rigid brain dynamics during cognitive performance. The optimal state for Task A is denoted as latent state A, and the optimal state for Task B is denoted as latent state B. **a** Stable and flexible dynamics: During task A, brain activity remains in latent state A. Upon switching to Task B, the brain transits efficiently from state A to state B, maintaining task-optimal dynamics. **b** Volatile dynamics: brain activity fails to stabilize in the task-optimal state, exhibiting frequent deviations from the optimal latent state for the current task to sub-optimal states. **c** Rigid dynamics: Following a switch from Task A to Task B, the brain remains persistently in latent state A, showing delayed disengagement and transition to the new task-optimal state B. **d** Schematic illustration of the state-space Bayesian Dynamic Switching System (BSDS) model: y_t_ denotes the observed fMRI timeseries at time point t; z_t_ denotes the latent states that are interdependent through a first order Markov chain; x_t_ denotes the latent space variables. Details of the model are described in a previous study [35]. **e** A previous study [68] has found that higher occupancy rates of the task-optimal brain state are associated with lower intra-individual response variability (IIRV) whereas higher occupancy rates of sub-optimal brain states are associated with higher IIRV in TD children and children with ADHD. **f** A previous study [31] has revealed that high occupancy rates of the task-optimal brain state are associated with better inhibitory control function (shorter SSRT) TD children and children with ADHD. Occupancy rate is calculated by dividing the number of time points assigned to a state by the total number of time points in the time series.

Cognition relies on the brain’s capacity to sustain both stable and flexible communication across large-scale networks, facilitating the timely engagement and maintenance of task-optimal brain states while resisting the intrusion of sub-optimal brain states (Fig. 5a) [30–36]. Disruptions in this dynamic balance, manifesting as excessive volatility (i.e., difficulty maintaining task-optimal brain states; Fig. 5b) or excessive rigidity (i.e., delayed disengagement from sub-optimal brain states; Fig. 5c), have been linked to cognitive instability and inflexibility [35, 68, 195]. In a seminal review [37], Sonuga-Barke and Castellanos proposed the default mode interference hypothesis, which posits that inadequate suppression of default mode network activity may allow task-irrelevant states to intrude during goal-directed cognitive processing. These intrusions can compete for limited cognitive resource, leading to attentional lapse and impaired performance in individuals with ADHD. This hypothesis provides a neurobiologically plausible account of the elevated IIRV, a behavioral hallmark in ADHD [69–71, 196], and is supported by neuroimaging evidence showing dysregulated default mode network activity during cognitive tasks and its association with IIRV [178, 197–199]. Consistent with the role of default mode network in moment-to-moment attentional fluctuation, Esterman et al. found that increased commission error rates were preceded by elevated default mode network activity, particularly when participants were in a stable performance state [72].

**Fig. 6:**
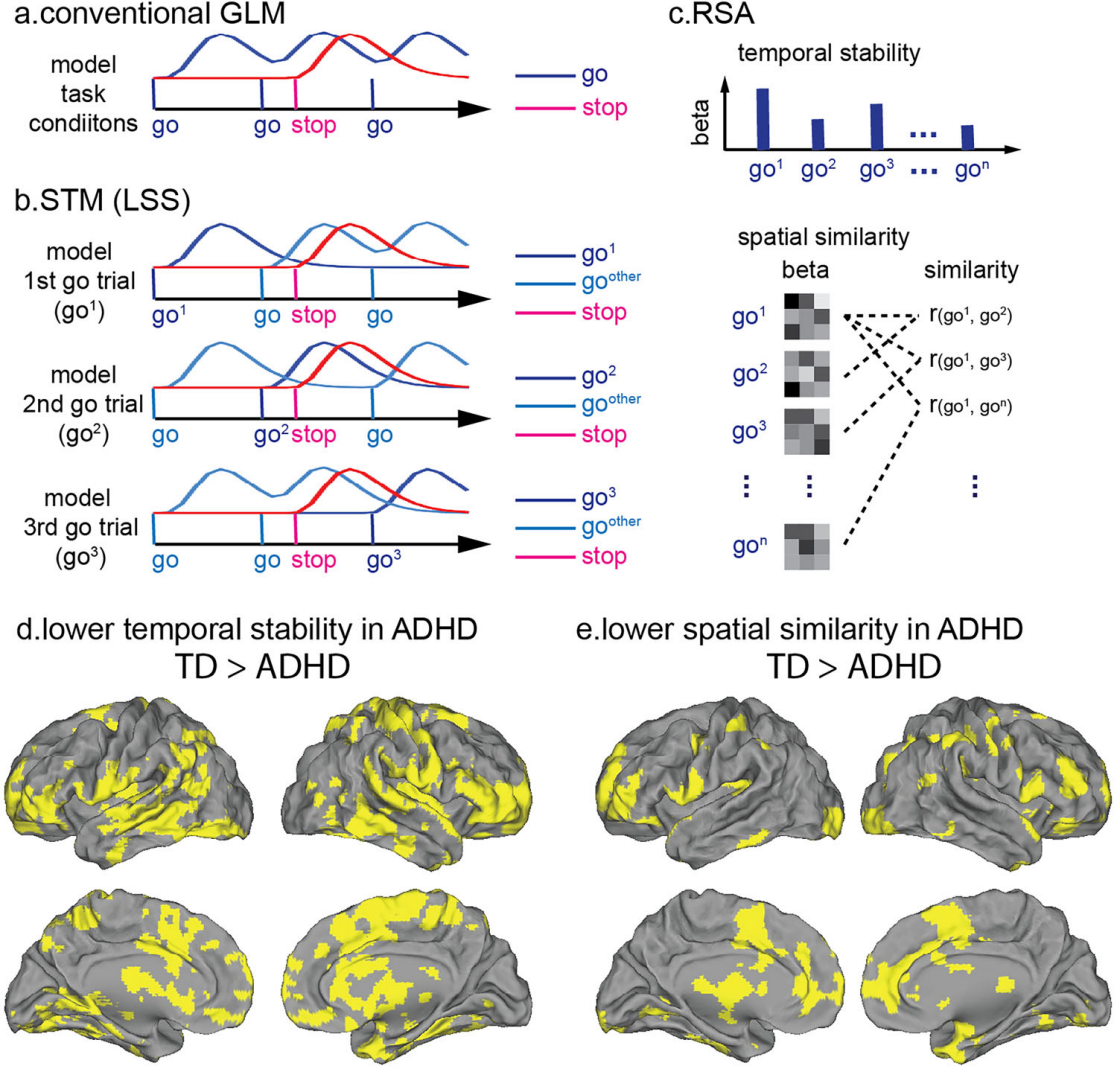
Single trial model (STM) and representational similarity analysis (RSA). **a** The conventional general linear model (GLM) estimates condition-specific brain activation pattern, i.e. task-evoked activation across all the go trials. **b** STM estimates trial-specific brain activation pattern. For example, in the STM for the 1^st^ go trial, the Least-Squares Separate (LSS) technique separate the vector of the 1^st^ go trial from the vector of the rest of the go trials and the vectors of other condition. The same approach is applied for estimation of activation pattern elicited by each go trial. **c** RSA can examine temporal stability and spatial similarity of trial-wise brain activation. A recent study has used STM + RSA methods to demonstrate children with ADHD have (**d**) lower temporal stability and (**e**) lower spatial similarity of trial-wise activation pattern than TD children (*p* < 0.05, TFCE corrected) during the conditional stop-signal task [108].

An open question is whether cognitive deficits in ADHD arise from difficulty in sustaining task-optimal states, increased susceptibility to sub-optimal state intrusion, or both, particularly within neural systems governing inhibitory control and motivation. The traditional general linear model (GLM), which estimates averaged condition-specific activation pattern (Fig. 6a), is incapable of capturing continuous fluctuation in neural activity during task performance, especially when such fluctuations are not tightly time-locked to experimental events. Moreover, model-driven analytic approaches, such as GLM, typically assume that similar behaviors are generated by similar underlying neural processes. However, this assumption often fails in practice. For instance, a slower response may result from attentional lapse, fatigue, or effortful proactive control, with distinct brain mechanisms producing superficially similar outcomes. As such, neurodynamic modeling approaches are essential for detecting ongoing changes in brain activity driven not only by experimental manipulation (e.g., cognitive demand) but also by non-experimental influences (e.g., practice effect, motivation, fatigue or attentional lapse).

Although widely applied in resting-state fMRI studies, the sliding window approach, which computes dynamic measures by moving a fixed-size window across the time series [200], is less suited for task-state fMRI research because of the difficulties in selecting an appropriate window size and the risk of crossing task structure boundaries. In contrast, state-space models have proven effective for uncovering latent brain dynamics that are not necessarily time-locked to task events [35, 201–204]. These models typically define states as unique patterns of brain activation and connectivity [205], and describe their temporal evolution in probabilistic frameworks such as hidden Markov models.

In state-space models, latent brain states are characterized by distinct spatial configurations of brain activation or functional connectivity. The temporal dynamics of these states are typically quantified using two key metrics: occupancy rate (or fractional occupancy) and mean lifetime (or dwell time). The occupancy rate represents the overall dominance of a state, defined as the proportion of the total task duration that an individual spends in that specific latent state. It is calculated by dividing the number of time points assigned to a state by the total number of time points in the time series. The mean lifetime reflects state stability, defined as the average continuous duration a state persists before transitioning to a different state.

Yamashita et al. used an energy landscape analysis to identify optimal and sub-optimal brain states during a sustained attention task [204]. Their findings showed that adults with ADHD spent more time in the sub-optimal state and less time in the optimal state compared to controls. However, the method relies on binarization of brain activation, which limits the interpretability of the resulting brain state features and may contribute to inconsistency in the findings. In another study, Cai et al. applied the state-space Bayesian Switch Dynamic System model (Fig. 5d) to identify latent brain states during a choice response task [68]. They found that more time spent in a task-optimal state is related to decreased IIRV whereas more time spent in sub-optimal states is associated with increased IIRV (Fig. 5e). Moreover, time spent in non-optimal state, the state characterized by weaker functional connectivity between regions in salience and frontoparietal networks relative to the task-optimal state, predicted inattention symptoms. Similarly, another study found that higher probabilities of engaging task-optimal brain state were linked to better inhibitory control function (i.e., faster SSRTs) and less severe inattention problem [31] (Fig. 5f). Together, these findings highlight aberrant brain dynamics in ADHD during cognitive performance and also demonstrate the utility of state-space modeling in revealing dynamic neural signatures of ADHD.

Despite the strengths of state-space modeling, aligning state-space-inferred dynamics with discrete task events can be challenging, particularly in fast event-related design. To better capture trial-wise neural dynamics, single-trial models (STMs) have emerged as a powerful alternative [206, 207] (Fig. 6b). When combined with representation similarity analysis (RSA), STMs enable the quantification of both activation magnitude and spatial pattern similarity across trials (Fig. 6c), which have been widely used to study memory, learning, pain and social function [208–211]. In a recent study, Gao et al. innovatively employed the STM-RSA framework to study dynamic dual control deficits in children with ADHD [108]. They developed two novel metrics to characterize temporal and spatial properties of brain dynamics: temporal and spatial stability. Temporal stability measures the consistency of activation strength across trials, whereas spatial stability quantifies the consistency of activation patterns among neighboring voxels across trials (Fig. 6c). They found that children with ADHD exhibit reduced temporal and spatial stability of task-evoked activation patterns during a conditional stop-signal task compared to TD children (Fig. 6d-e) [108]. Notably, although trial-by-trial variation in RT was tracked by activation fluctuations in key inhibitory control regions, such as anterior insula and pre-supplementary motor area, in TD children, this relation was markedly attenuated in children with ADHD [108]. These findings highlight not only diminished stability in neural activity but also compromised neurocognitive mechanisms underlying trial-wise response modulation in ADHD.

By leveraging advanced computational approaches such as state-space modeling and single-trial models, neurodynamic frameworks can offer novel insights into the brain mechanisms underlying cognitive deficits associated with ADHD.

## Pharmacological effect on cognitive control and reward systems

ADHD medications, both stimulant and non-stimulant, are widely prescribed to alleviate core clinical symptoms of the disorder [212, 213]. A meta-analysis has shown that these treatments can have a protective effect across various domains of daily functioning, ranging from academic performance to road accidents [214]. However, therapeutic response is heterogeneous, with some individuals showing minimal benefit [215, 216]. This variability is likely to reflect underlying neurobiological differences, underscoring the need for biomarkers that can predict individual treatment response.

In line with cognitive models of ADHD, first-line stimulant medications have been consistently shown to enhance inhibitory control functions in affected individuals [217–219]. For instance, a recent study demonstrated that a single dose of methylphenidate improved inhibition accuracy and reduced behavioral variability in children with ADHD [220, 221]. Meta-analytic findings support these results, revealing that methylphenidate significantly improves performance on response inhibition tasks, such as stop-signal and go/no-go paradigms [217–219].

Beyond inhibitory control, stimulant medication may also enhance motivation. For example, amphetamine treatment has been shown to increase willingness to exert physical efforts [130], and methylphenidate has been found to enhance engagement in effortful tasks in adults with ADHD [129]. Additional behavioral studies report that methylphenidate increases reward sensitivity, reduces delay discounting, and decreases effort-related avoidance [222–224]. These findings suggest that stimulants can promote goal-directed behavior, particularly in tasks requiring sustained effort or delayed gratification. Nonetheless, motivational and reward-related effects have been less systematically studied than cognitive outcomes.

Neuropharmacological imaging studies provide mechanistic insights into these effects. Methylphenidate has been shown to modulate activity in brain regions implicated in both inhibitory control and reward processing, including the anterior insula, anterior cingulate cortex, and striatum [225, 226]. Although relatively few neuroimaging studies have investigated medication-induced changes in brain activation and connectivity during inhibitory control and reward processing tasks in children with ADHD [98, 178, 227, 228], a meta-analysis revealed that stimulant reliably enhances activation in the bilateral inferior frontal cortex and anterior insula during successful inhibition compared to placebo [229]. These findings support a neuropharmacological basis for the cognitive improvements observed with stimulant treatment.

At a cellular level, stimulant medications such as methylphenidate and amphetamine exert their effects by increasing extracellular dopamine and norepinephrine levels in the brain [230, 231]. Methylphenidate achieves this by blocking their respective transporters and inhibiting reuptake [232, 233]. Amphetamines not only inhibit reuptake but also promote presynaptic release of these catecholamines [234]. These mechanisms are thought to enhance the efficiency of frontostriatal and prefrontal cortical circuits involved in cognitive control, and likely contribute to increased reward sensitivity through their effects on mesolimbic dopamine pathways, key systems for motivation, reward anticipation, and reinforcement learning [118, 235].

In contrast, non-stimulant medications such as atomoxetine and guanfacine exert their effects through different neurochemical mechanisms. Atomoxetine is a selective norepinephrine reuptake inhibitor that primarily increases norepinephrine levels in the prefrontal cortex by blocking the norepinephrine transporter, thereby enhancing attention and inhibitory control [236–238]. Guanfacine, an α2A-adrenergic receptor agonist, enhances prefrontal cortical network regulation by inhibiting cAMP signaling, stabilizing neuronal firing, and supporting circuits underlying attention and working memory [239–241]. This suggests that stimulant medications may have broader effects encompassing both cognitive control and reward systems, whereas non-stimulants primarily target prefrontal executive dysfunction.

Recent research has begun to uncover how methylphenidate modulates atypical brain dynamics in children with ADHD, offering promising bridges to clinical application. For example, Mizuno et al. demonstrated that methylphenidate reduces aberrant dynamic interactions among the salience, frontoparietal, and default mode networks, effectively alleviating atypical dynamic network interactions in children with ADHD [221]. These findings were further replicated using advanced brain dynamic modelling approaches, reinforcing the robustness of this remediation effect [242]. Additionally, methylphenidate was shown to enhance dynamic neural activity in the nucleus accumbens, a key node in the reward circuitry [220]. Critically, these medication-induced alterations in the inhibitory control and reward systems were associated with corresponding improvements in inhibitory control performance, suggesting that changes in brain state dynamics translate to behavioral gains [220, 221, 242]. These findings revealed novel neurodynamic mechanisms of methylphenidate, offering circuit-level insights that can inform precision psychiatry.

## Limitations, challenges and future research

Emerging research increasingly leverages computational models to characterize the dynamic latent cognitive and neural processes that underlie cognitive functioning. These models hold significant promise for uncovering abnormality in neurocognitive dynamics. By capturing fine-grained, time-resolved patterns of brain activity and behavior, computational frameworks such as state-space and single-trial models can offer mechanistic insights into the temporal dynamics of cognitive processes in ADHD.

Motivational deficit is mostly assessed through self-report and investigated using reward-dependent action-outcome contingency in experimental setting. However, these approaches serve only as indirect proxies rather than direct, quantitative measures of motivation, limiting mechanistic understanding of motivational deficits. Future effort should focus on developing novel experimental paradigms and computational models to accurately characterize human motivation [243, 244], potentially leveraging insights from advancements in animal research [245].

Neuroimaging research in ADHD faces several significant challenges. A key issue is the high drop-out rate, largely due to the young age and excessive head motion. Earlier studies were limited by small sample sizes. Large-scale neuroimaging initiatives, e.g. ABCD, HCP-D and IMAGEN, have begun to address this limitation. Furthermore, children with more severe symptoms are more likely to be excluded, leading to sampling bias. Although it is acknowledged in the field, a clear solution has yet to emerge. One potential avenue is the dimensional analysis, which may offer a way to mitigate bias by focusing on symptom continua.

Another major challenge is the intervention effect. A common approach is to implement a washout period for stimulant medications; however, the long-term effects of these treatments remain under investigation. Washout periods for non-stimulant medicines are longer and less feasible, further complicating study design. In recent years, neurostimulation techniques have emerged as promising new intervention options. However, these methods are still in their early stage and lack standardized parameters. Developing appropriate washout protocols for participants undergoing neurostimulation remains an open question.

No studies have systematically investigated how non-stimulant medications influence large-scale brain dynamics in children with ADHD. A deeper understanding of how different pharmacological agents modulate brain function could inform precision psychiatry, enabling treatment to be tailored to individuals’ neurobiological profiles.

## Conclusion

Decades of behavioral and neuroimaging research have provided substantial evidence of impairments in both inhibitory control and motivation/reward systems in children with ADHD. However, the precise nature of these disrupted neural mechanisms and how they contribute to specific behavioral problems still remains poorly understood. We propose incorporating dynamic perspectives into existing neurocognitive models to examine how task-dependent brain dynamics influence downstream cognitive functioning and dysfunction in children with ADHD. Further research is critically needed to elucidate the dynamic interplay between motivation/reward and inhibitory control systems, which could yield valuable insights into the underlying heterogeneity of the disorder and developing effective and personalized interventions.

## References

[1] Biederman J, Faraone SV. Attention-deficit hyperactivity disorder. Lancet. 2005;366:237–48. 10.1016/S0140-6736(05)66915-2. 16023516 10.1016/S0140-6736(05)66915-2

[2] Faraone SV, Biederman J. What is the prevalence of adult ADHD? Results of a population screen of 966 adults. J Atten Disord. 2005;9:384–91. 10.1177/1087054705281478. 16371661 10.1177/1087054705281478

[3] Faraone SV, Sergeant J, Gillberg C, Biederman J. The worldwide prevalence of ADHD: is it an American condition?. World psychiatry. 2003;2:104–13. 16946911 PMC1525089

[4] Mick E, Faraone SV, Biederman J. Age-dependent expression of attention-deficit/hyperactivity disorder symptoms. Psychiatr Clin North Am. 2004;27:215–24. 10.1016/j.psc.2004.01.003. 15063994 10.1016/j.psc.2004.01.003

[5] Polanczyk G, de Lima MS, Horta BL, Biederman J, Rohde LA. The worldwide prevalence of ADHD: a systematic review and metaregression analysis. Am J psychiatry. 2007;164:942–8. 10.1176/appi.ajp.164.6.942. 17541055 10.1176/ajp.2007.164.6.942

[6] Getahun D, Jacobsen SJ, Fassett MJ, Chen W, Demissie K, Rhoads GG. Recent trends in childhood attention-deficit/hyperactivity disorder. Jama Pediatrics. 2013;167:282–8. 10.1001/2013.jamapediatrics.401. 23338799 10.1001/2013.jamapediatrics.401

[7] Xu G, Strathearn L, Liu B, Yang B, Bao W. Twenty-year trends in diagnosed attention-deficit/hyperactivity disorder among US children and adolescents, 1997-2016. JAMA Pediatrics. 2018;1:e181471. 10.1001/jamanetworkopen.2018.1471 PMC6324288 30646132

[8] Danielson ML, Claussen AH, Bitsko RH, Katz SM, Newsome K, Blumberg SJ, et al. ADHD Prevalence Among U.S. Children and Adolescents in 2022: Diagnosis, Severity, Co-Occurring Disorders, and Treatment. J Clin child Adolesc Psychol. 2024;53:343–60. 10.1080/15374416.2024.2335625. 38778436 10.1080/15374416.2024.2335625 PMC11334226

[9] Hinshaw SP. Attention deficit hyperactivity disorder (ADHD): controversy, developmental mechanisms, and multiple levels of analysis. Annu Rev Clin Psychol. 2018;14:291–316. 10.1146/annurev-clinpsy-050817084917. 29220204 10.1146/annurev-clinpsy-050817-084917

[10] Dalrymple RA, Maxwell LM, Russell S, Duthie J. NICE guideline review: attention deficit hyperactivity disorder: diagnosis and management (NG87). Arch Dis Childhood-E. 2020;105:289–93. 10.1136/archdischild-2019-316928. 10.1136/archdischild-2019-316928 31776172

[11] Wolraich ML, Hagan JF Jr, Allan C, Chan E, Davison D, Earls M, et al. Clinical practice guideline for the diagnosis, evaluation, and treatment of attention-deficit/hyperactivity disorder in children and adolescents. Pediatrics. 2019;144:e20192528. 31570648 10.1542/peds.2019-2528 PMC7067282

[12] Barkley RA. Behavioral inhibition, sustained attention, and executive functions: constructing a unifying theory of ADHD. Psychological Bull. 1997;121:65–94. 10.1037/0033-2909.121.1.65 9000892

[13] Castellanos FX, Sonuga-Barke EJ, Milham MP, Tannock R. Characterizing cognition in ADHD: beyond executive dysfunction. Trends Cognit Sci. 2006;10:117–23. 10.1016/j.tics.2006.01.011. 16460990 10.1016/j.tics.2006.01.011

[14] Castellanos FX, Tannock R. Neuroscience of attention-deficit/hyperactivity disorder: the search for endophenotypes. Nat reviews Neurosci. 2002;3:617–28. 10.1038/nrn896. 10.1038/nrn896 12154363

[15] Haenlein M, Caul WF. Attention-Deficit disorder with hyperactivity - a specific hypothesis of reward dysfunction. J Am Acad Child Adolesc Psychiatry. 1987;26:356–62. 10.1097/00004583-198705000-00014. 3597291 10.1097/00004583-198705000-00014

[16] Nigg JT. Is ADHD a disinhibitory disorder?. Psychological Bull. 2001;127:571–98. 10.1037/0033-2909.127.5.571 11548968

[17] Nigg JT, Casey BJ. An integrative theory of attention-deficit/ hyperactivity disorder based on the cognitive and affective neurosciences. Dev psychopathology. 2005;17:785–806. 10.1017/S0954579405050376. 10.1017/S0954579405050376 16262992

[18] Nigg JT, Goldsmith HH, Sachek J. Temperament and attention deficit hyperactivity disorder: the development of a multiple pathway model. J Clin child Adolesc Psychol. 2004;33:42–53. 10.1207/S15374424JCCP3301_5. 15028540 10.1207/S15374424JCCP3301_5

[19] Sagvolden T, Aase H, Zeiner P, Berger D. Altered reinforcement mechanisms in attention-deficit/hyperactivity disorder. Behav Brain Res. 1998;94:61–71. 9708840

[20] Sonuga-Barke EJ. Causal models of attention-deficit/hyperactivity disorder: from common simple deficits to multiple developmental pathways. Biol Psychiatry. 2005;57:1231–8. 10.1016/j.biopsych.2004.09.008. 15949993 10.1016/j.biopsych.2004.09.008

[21] Sonuga-Barke EJS, Bitsakou P, Thompson M. Beyond the dual pathway model: evidence for the dissociation of timing, inhibitory, and delay-related impairments in attention-deficit/hyperactivity disorder. J Am Acad Child Adolesc Psychiatry. 2010;49:345–55. 10.1016/j.jaac.2009.12.018. 20410727 10.1016/j.jaac.2009.12.018

[22] Botvinick M, Braver T. Motivation and cognitive control: from behavior to neural mechanism. Annu Rev Psychol. 2015;66:83–113. 10.1146/annurev-psych-010814-015044. 25251491 10.1146/annurev-psych-010814-015044

[23] Parro C, Dixon ML, Christoff K. The neural basis of motivational influences on cognitive control. Hum brain Mapp. 2018;39:5097–111. 10.1002/hbm.24348. 30120846 10.1002/hbm.24348 PMC6866502

[24] Marx I, Hacker T, Yu X, Cortese S, Sonuga-Barke E. ADHD and the choice of small immediate over larger delayed rewards: a comparative meta-analysis of performance on simple choice-delay and temporal discounting paradigms. J Atten Disord. 2021;25:171–87. 10.1177/1087054718772138. 29806533 10.1177/1087054718772138

[25] Willcutt EG, Doyle AE, Nigg JT, Faraone SV, Pennington BF. Validity of the executive function theory of attention-deficit/hyperactivity disorder: a meta-analytic review. Biol Psychiatry. 2005;57:1336–46. 10.1016/j.biopsych.2005.02.006. 15950006 10.1016/j.biopsych.2005.02.006

[26] Hart H, Radua J, Nakao T, Mataix-Cols D, Rubia K. Meta-analysis of functional magnetic resonance imaging studies of inhibition and attention in attention-deficit/hyperactivity disorder: exploring task-specific, stimulant medication, and age effects. JAMA psychiatry. 2013;70:185–98. 10.1001/jamapsychiatry.2013.277. 23247506 10.1001/jamapsychiatry.2013.277

[27] Posner J, Park C, Wang Z. Connecting the dots: a review of resting connectivity MRI studies in attention-deficit/hyperactivity disorder. Neuropsychology Rev. 2014;24:3–15. 10.1007/s11065-014-9251-z. 10.1007/s11065-014-9251-z PMC4119002 24496902

[28] Cortese S, Aoki YY, Itahashi T, Castellanos FX, Eickhoff SB. Systematic review and meta-analysis: resting-state functional magnetic resonance imaging studies of attention-deficit/hyperactivity disorder. J Am Acad Child Adolesc Psychiatry. 2021;60:61–75. 10.1016/j.jaac.2020.08.014. 32946973 10.1016/j.jaac.2020.08.014

[29] Samea F, Soluki S, Nejati V, Zarei M, Cortese S, Eickhoff SB, et al. Brain alterations in children/adolescents with ADHD revisited: a neuroimaging meta-analysis of 96 structural and functional studies. Neurosci Biobehav Rev. 2019;100:1–8. 10.1016/j.neubiorev.2019.02.011. 30790635 10.1016/j.neubiorev.2019.02.011 PMC7966818

[30] Braun U, Schäfer A, Walter H, Erk S, Romanczuk-Seiferth N, Haddad L, et al. Dynamic reconfiguration of frontal brain networks during executive cognition in humans. Proc Natl Acad Sci USA. 2015;112:11678–83. 10.1073/pnas.1422487112. 26324898 10.1073/pnas.1422487112 PMC4577153

[31] Cai WD, Taghia J, Menon V. A multi-demand operating system underlying diverse cognitive tasks. Nat Commun. 2024;15:2185. 10.1038/s41467-024-46511-5. 38467606 10.1038/s41467-024-46511-5 PMC10928152

[32] Kitzbichler MG, Henson RNA, Smith ML, Nathan PJ, Bullmore ET. Cognitive effort drives workspace configuration of human brain functional networks. J Neurosci. 2011;31:8259–70. 10.1523/Jneurosci.0440-11.2011. 21632947 10.1523/JNEUROSCI.0440-11.2011 PMC6622866

[33] Shine JM, Bissett PG, Bell PT, Koyejo O, Balsters JH, Gorgolewski KJ, et al. The dynamics of functional brain networks: integrated network states during cognitive task performance. Neuron. 2016;92:544–54. 10.1016/j.neuron.2016.09.018. 27693256 10.1016/j.neuron.2016.09.018 PMC5073034

[34] Spadone S, Della Penna S, Sestieri C, Betti V, Tosoni A, Perrucci MG, et al. Dynamic reorganization of human resting-state networks during visuospatial attention. Proc Natl Acad Sci USA. 2015;112:8112–7. 10.1073/pnas.1415439112. 26080395 10.1073/pnas.1415439112 PMC4491799

[35] Taghia J, Cai W, Ryali S, Kochalka J, Nicholas J, Chen T, et al. Uncovering hidden brain state dynamics that regulate performance and decision-making during cognition. Nat Commun. 2018;9:2505. 29950686 10.1038/s41467-018-04723-6 PMC6021386

[36] Vatansever D, Menon DK, Manktelow AE, Sahakian BJ, Stamatakis EA. Default mode dynamics for global functional integration. J Neurosci. 2015;35:15254–62. 10.1523/Jneurosci.2135-15.2015. 26586814 10.1523/JNEUROSCI.2135-15.2015 PMC4649001

[37] Sonuga-Barke EJ, Castellanos FX. Spontaneous attentional fluctuations in impaired states and pathological conditions: a neurobiological hypothesis. Neurosci Biobehav Rev. 2007;31:977–86. 10.1016/j.neubiorev.2007.02.005. 17445893 10.1016/j.neubiorev.2007.02.005

[38] Schachar R, Logan GD. Impulsivity and inhibitory control in normal development and childhood psychopathology. Developmental Psychol. 1990;26:710–20. 10.1037/0012-1649.26.5.710.

[39] Williams BR, Ponesse JS, Schachar RJ, Logan GD, Tannock R. Development of inhibitory control across the life span. Developmental Psychol. 1999;35:205–13. 10.1037//0012-1649.35.1.205 9923475

[40] Cai W, Duberg K, Padmanabhan A, Rehert R, Bradley T, Carrion V, et al. Hyperdirect insula-basal ganglia pathway and adult-like maturity of global brain responses predict inhibitory control in children. Nat Commun. 2019;10:1–13. 31641118 10.1038/s41467-019-12756-8 PMC6805945

[41] Association, AP (Washington D.C., 2013).

[42] Cai W, Oldenkamp CL, Aron AR. Stopping speech suppresses the task-irrelevant hand. Brain Lang. 2012;120:412–5. 10.1016/j.bandl.2011.11.006. 22206872 10.1016/j.bandl.2011.11.006 PMC3533487

[43] Leung HC, Cai W. Common and differential ventrolateral prefrontal activity during inhibition of hand and eye movements. J Neurosci. 2007;27:9893–9900. 10.1523/JNEUROSCI.2837-07.2007. 17855604 10.1523/JNEUROSCI.2837-07.2007 PMC6672638

[44] Tatz JR, Soh C, Wessel JR. Common and unique inhibitory control signatures of action-stopping and attentional capture suggest that actions are stopped in two stages. J Neurosci. 2021;41:8826–38. 10.1523/Jneurosci.1105-21.2021. 34493541 10.1523/JNEUROSCI.1105-21.2021 PMC8528501

[45] Wessel JR, Anderson MC. Neural mechanisms of domain-general inhibitory control. Trends Cognit Sci. 2024;28:124–43. 10.1016/j.tics.2023.09.008. 10.1016/j.tics.2023.09.008 PMC13386798 39492255

[46] Logan GD, Cowan WB, Davis KA. On the ability to inhibit simple and choice reaction time responses: a model and a method. J Exp psychology Hum Percept Perform. 1984;10:276–91. 10.1037//0096-1523.10.2.276 6232345

[47] Verbruggen F, Logan GD. Response inhibition in the stop-signal paradigm. Trends Cognit Sci. 2008;12:418–24. 10.1016/j.tics.2008.07.005. 18799345 10.1016/j.tics.2008.07.005 PMC2709177

[48] Hanes DP, Patterson WF 2nd, Schall JD. Role of frontal eye fields in countermanding saccades: visual, movement, and fixation activity. J Neurophysiol. 1998;79:817–34. 10.1152/jn.1998.79.2.817. 9463444 10.1152/jn.1998.79.2.817

[49] Schall JD. The neural selection and control of saccades by the frontal eye field. Philos T Roy Soc B. 2002;357:1073–82. 10.1098/rstb.2002.1098. 10.1098/rstb.2002.1098 PMC1693021 12217175

[50] Cai W, Warren SL, Duberg K, Yu A, Hinshaw SP, Menon V. Both reactive and proactive control are deficient in children with ADHD and predictive of clinical symptoms. Transl Psychiatry. 2023;13:179. 37236924 10.1038/s41398-023-02471-w PMC10220086

[51] Janssen TWP, Heslenfeld DJ, van Mourik R, Logan GD, Oosterlaan J. Neural correlates of response inhibition in children with attention-deficit/hyperactivity disorder: a controlled version of the stop-signal task. Psychiat Res-Neuroim. 2015;233:278–84. 10.1016/j.pscychresns.2015.07.007. 10.1016/j.pscychresns.2015.07.007 26195296

[52] Tillman CM, Thorell LB, Brocki KC, Bohlin G. Motor response inhibition and execution in the stop-signal task: Development and relation to ADHD behaviors. Child Neuropsychol. 2008;14:42–59. 10.1080/09297040701249020. 17852128 10.1080/09297040701249020

[53] Aron AR. From reactive to proactive and selective control: developing a richer model for stopping inappropriate responses. Biol Psychiatry. 2011;69:e55–68. 10.1016/j.biopsych.2010.07.024. 20932513 10.1016/j.biopsych.2010.07.024 PMC3039712

[54] Braver TS. The variable nature of cognitive control: a dual mechanisms framework. Trends Cognit Sci. 2012;16:106–13. 10.1016/j.tics.2011.12.010. 22245618 10.1016/j.tics.2011.12.010 PMC3289517

[55] Chikazoe J, Jimura K, Hirose S, Yamashita K, Miyashita Y, Konishi S. Preparation to inhibit a response complements response inhibition during performance of a stop-signal task. J Neurosci. 2009;29:15870–7. 10.1523/JNEUROSCI.3645-09.2009. 20016103 10.1523/JNEUROSCI.3645-09.2009 PMC6666181

[56] Cai W, Oldenkamp CL, Aron AR. A proactive mechanism for selective suppression of response tendencies. J Neurosci. 2011;31:5965–9. 10.1523/JNEUROSCI.6292-10.2011. 21508221 10.1523/JNEUROSCI.6292-10.2011 PMC3111595

[57] Majid DS, Cai W, Corey-Bloom J, Aron AR. Proactive Selective Response Suppression Is Implemented via the Basal Ganglia. J Neurosci. 2013;33:13259–69. 10.1523/JNEUROSCI.5651-12.2013. 23946385 10.1523/JNEUROSCI.5651-12.2013 PMC3742918

[58] Swann NC, Cai W, Conner CR, Pieters TA, Claffey MP, George JS, et al. Roles for the pre-supplementary motor area and the right inferior frontal gyrus in stopping action: electrophysiological responses and functional and structural connectivity. NeuroImage. 2012;59:2860–70. 10.1016/j.neuroimage.2011.09.049. 21979383 10.1016/j.neuroimage.2011.09.049 PMC3322194

[59] Chatham CH, Frank MJ, Munakata Y. Pupillometric and behavioral markers of a developmental shift in the temporal dynamics of cognitive control. Proc Natl Acad Sci USA. 2009;106:5529–33. 10.1073/pnas.0810002106. 19321427 10.1073/pnas.0810002106 PMC2666994

[60] Chevalier N, Martis SB, Curran T, Munakata Y. Metacognitive processes in executive control development: the case of reactive and proactive control. J Cognit Neurosci. 2015;27:1125–36. 10.1162/jocn_a_00782. 25603026 10.1162/jocn_a_00782 PMC4510990

[61] Chevalier N, Meaney JA, Traut HJ, Munakata Y. Adaptiveness in proactive control engagement in children and adults. Developmental Cognit Neurosci. 2020;46:100870. 10.1016/j.dcn.2020.100870. 10.1016/j.dcn.2020.100870 PMC7591345 33120165

[62] Gonthier C, Zira M, Colé P, Blaye A. Evidencing the developmental shift from reactive to proactive control in early childhood and its relationship to working memory. J Exp Child Psychol. 2019;177:1–16. 10.1016/j.jecp.2018.07.001. 30165288 10.1016/j.jecp.2018.07.001

[63] Iselin AM, Decoster J. Reactive and proactive control in incarcerated and community adolescents and young adults. Cognit Dev. 2009;24:192–206. 10.1016/j.cogdev.2008.07.001. 20161210 10.1016/j.cogdev.2008.07.001 PMC2714918

[64] Pani P, Menghini D, Napolitano C, Calcagni M, Armando M, Sergeant JA, et al. Proactive and reactive control of movement are differently affected in attention deficit hyperactivity disorder children. Res developmental disabilities. 2013;34:3104–11. 10.1016/j.ridd.2013.06.032. 10.1016/j.ridd.2013.06.032 23886755

[65] van Hulst BM, de Zeeuw P, Vlaskamp C, Rijks Y, Zandbelt BB, Durston S. Children with ADHD symptoms show deficits in reactive but not proactive inhibition, irrespective of their formal diagnosis. Psychological Med. 2018;48:2515–21. 10.1017/S0033291718000107. 10.1017/S0033291718000107 PMC6190063 29415788

[66] Heathcote A, Popiel SJ, Mewhort DJK. Analysis of response-time distributions - an example using the stroop task. Psychological Bull. 1991;109:340–7. 10.1037/0033-2909.109.2.340.

[67] Ratcliff R, Murdock BB. Retrieval Processes in Recognition Memory. Psychol Rev. 1976;83:190–214. 10.1037/0033-295x.83.3.190.

[68] Cai W, Warren SL, Duberg K, Pennington B, Hinshaw SP, Menon V. Latent brain state dynamics distinguish behavioral variability, impaired decision-making, and inattention. Mol Psychiatry. 2021;26:4944–57. 10.1038/s41380-021-01022-3. 33589738 10.1038/s41380-021-01022-3 PMC8589642

[69] Klein C, Wendling K, Huettner P, Ruder H, Peper M. Intra-subject variability in attention-deficit hyperactivity disorder. Biol Psychiatry. 2006;60:1088–97. 10.1016/j.biopsych.2006.04.003. 16806097 10.1016/j.biopsych.2006.04.003

[70] Kofler MJ, Rapport MD, Sarver DE, Raiker JS, Orban SA, Friedman LM, et al. Reaction time variability in ADHD: a meta-analytic review of 319 studies. Clin Psychol Rev. 2013;33:795–811. 10.1016/j.cpr.2013.06.001. 23872284 10.1016/j.cpr.2013.06.001

[71] Kuntsi J, Klein C. Intraindividual variability in ADHD and its implications for research of causal links. Curr Top Behav Neurosci. 2012;9:67–91. 10.1007/7854_2011_145. 21769722 10.1007/7854_2011_145

[72] Esterman M, Noonan SK, Rosenberg M, Degutis J. In the zone or zoning out? Tracking behavioral and neural fluctuations during sustained attention. Cereb cortex. 2013;23:2712–23. 10.1093/cercor/bhs261. 22941724 10.1093/cercor/bhs261

[73] Wang C, Ding MZ, Kluger BM. Change in intraindividual variability over time as a key metric for defining performance-based cognitive fatigability. Brain Cognition. 2014;85:251–8. 10.1016/j.bandc.2014.01.004. 24486386 10.1016/j.bandc.2014.01.004 PMC3980793

[74] Mistry, P, Branigan, NK, Gao, Z, Cai, W & Menon, V Computational modeling of proactive, reactive, and attentional dynamics in cognitive control. bioRxiv [Preprint]. 2024. Available from: https://www.biorxiv.org/content/10.1101/2024.10.01.615613v1

[75] Shenoy P, Yu AJ. Rational decision-making in inhibitory control. Front Hum Neurosci. 2011;5:48. 10.3389/fnhum.2011.00048. 21647306 10.3389/fnhum.2011.00048 PMC3103997

[76] Cai WD, Chen TW, Ide JS, Li CSR, Menon V. Dissociable fronto-operculum-insula control signals for anticipation and detection of inhibitory sensory cue. Cereb cortex. 2017;27:4073–82. 10.1093/cercor/bhw219. 27473319 10.1093/cercor/bhw219 PMC6059112

[77] Ide JS, Shenoy P, Yu AJ, Li CS. Bayesian prediction and evaluation in the anterior cingulate cortex. J Neurosci. 2013;33:2039–47. 10.1523/JNEUROSCI.2201-12.2013. 23365241 10.1523/JNEUROSCI.2201-12.2013 PMC3711643

[78] Cai W, Ryali S, Chen T, Li CS, Menon V. Dissociable roles of right inferior frontal cortex and anterior insula in inhibitory control: evidence from intrinsic and task-related functional parcellation, connectivity, and response profile analyses across multiple datasets. J Neurosci. 2014;34:14652–67. 10.1523/JNEUROSCI.3048-14.2014. 25355218 10.1523/JNEUROSCI.3048-14.2014 PMC4212065

[79] Swick D, Ashley V, Turken U. Are the neural correlates of stopping and not going identical? Quantitative meta-analysis of two response inhibition tasks. NeuroImage. 2011;56:1655–65. 21376819 10.1016/j.neuroimage.2011.02.070

[80] Alexander GE, Crutcher MD. Functional architecture of basal ganglia circuits - neural substrates of parallel processing. Trends Neurosci. 1990;13:266–71. 10.1016/0166-2236(90)90107-L. 1695401 10.1016/0166-2236(90)90107-l

[81] Nambu A. Seven problems on the basal ganglia. Curr Opin Neurobiol. 2008;18:595–604. 10.1016/j.conb.2008.11.001. 19081243 10.1016/j.conb.2008.11.001

[82] Nambu A, Tokuno H, Takada M. Functional significance of the cortico-subthalamo-pallidal ‘hyperdirect’ pathway. Neurosci Res. 2002;43:111–7. 10.1016/S0168-0102(02)00027-5. 12067746 10.1016/s0168-0102(02)00027-5

[83] Aron AR, Poldrack RA. Cortical and subcortical contributions to Stop signal response inhibition: role of the subthalamic nucleus. J Neurosci. 2006;26:2424–33. 10.1523/JNEUROSCI.4682-05.2006. 16510720 10.1523/JNEUROSCI.4682-05.2006 PMC6793670

[84] Chen W, de Hemptinne C, Miller AM, Leibbrand M, Little SJ, Lim DA, et al. Prefrontal-Subthalamic hyperdirect pathway modulates movement inhibition in humans. Neuron. 2020;106:579–588.e3. 10.1016/j.neuron.2020.02.012. 32155442 10.1016/j.neuron.2020.02.012 PMC7274135

[85] Forstmann BU, Keuken MC, Jahfari S, Bazin PL, Neumann J, Schäfer A, et al. Cortico-subthalamic white matter tract strength predicts interindividual efficacy in stopping a motor response. NeuroImage. 2012;60:370–5. 10.1016/j.neuroimage.2011.12.044. 22227131 10.1016/j.neuroimage.2011.12.044

[86] Polyakova Z, Chiken S, Hatanaka N, Nambu A. Cortical control of subthalamic neuronal activity through the hyperdirect and indirect pathways in monkeys. J Neurosci. 2020;40:7451–63. 10.1523/Jneurosci.0772-20.2020. 32847963 10.1523/JNEUROSCI.0772-20.2020 PMC7511188

[87] Harsay HA, Cohen MX, Oosterhof NN, Forstmann BU, Mars RB, Ridderinkhof KR. Functional connectivity of the striatum links motivation to action control in humans. J Neurosci. 2011;31:10701–11. 10.1523/Jneurosci.5415-10.2011. 21775613 10.1523/JNEUROSCI.5415-10.2011 PMC6622616

[88] Jahfari S, Waldorp L, van den Wildenberg WP, Scholte HS, Ridderinkhof KR, Forstmann BU. Effective connectivity reveals important roles for both the hyperdirect (fronto-subthalamic) and the indirect (fronto-striatal-pallidal) fronto-basal ganglia pathways during response inhibition. J Neurosci. 2011;31:6891–9. 10.1523/JNEUROSCI.5253-10.2011. 21543619 10.1523/JNEUROSCI.5253-10.2011 PMC6632844

[89] Rae CL, Hughes LE, Anderson MC, Rowe JB. The prefrontal cortex achieves inhibitory control by facilitating subcortical motor pathway connectivity. J Neurosci. 2015;35:786–94. 10.1523/JNEUROSCI.3093-13.2015. 25589771 10.1523/JNEUROSCI.3093-13.2015 PMC4293423

[90] Tsvetanov KA, Ye Z, Hughes L, Samu D, Treder MS, Wolpe N, et al. Activity and connectivity differences underlying inhibitory control across the adult life span. J Neurosci. 2018;38:7887–7900. 10.1523/Jneurosci.2919-17.2018. 30049889 10.1523/JNEUROSCI.2919-17.2018 PMC6125816

[91] Vink M, Zandbelt BB, Gladwin T, Hillegers M, Hoogendam JM, van den Wildenberg WP, et al. Frontostriatal activity and connectivity increase during proactive inhibition across adolescence and early adulthood. Hum brain Mapp. 2014;35:4415–27. 10.1002/hbm.22483. 24532023 10.1002/hbm.22483 PMC6869143

[92] Bhaijiwala M, Chevrier A, Schachar R. Withholding and canceling a response in ADHD adolescents. Brain Behav. 2014;4:602–14. 10.1002/brb3.244. 25328838 10.1002/brb3.244 PMC4086366

[93] Booth JR, Burman DD, Meyer JR, Lei Z, Trommer BL, Davenport ND, et al. Larger deficits in brain networks for response inhibition than for visual selective attention in attention deficit hyperactivity disorder (ADHD). J Child Psychol Psyc. 2005;46:94–111. 10.1111/j.1469-7610.2004.00337.x. 10.1111/j.1469-7610.2004.00337.x 15660647

[94] Durston S, Mulder M, Casey BJ, Ziermans T, van Engeland H. Activation in ventral prefrontal cortex is sensitive to genetic vulnerability for attention-deficit hyperactivity disorder. Biol Psychiat. 2006;60:1062–70. 10.1016/j.biopsych.2005.12.020. 16712804 10.1016/j.biopsych.2005.12.020

[95] Hart H, Chantiluke K, Cubillo AI, Smith AB, Simmons A, Brammer MJ, et al. Pattern classification of response inhibition in ADHD: Toward the development of neurobiological markers for ADHD. Hum brain Mapp. 2014;35:3083–94. 10.1002/hbm.22386. 24123508 10.1002/hbm.22386 PMC4190683

[96] Langer N, Benjamin C, Becker BLC, Gaab N. Comorbidity of reading disabilities and ADHD: Structural and functional brain characteristics. Hum brain Mapp. 2019;40:2677–98. 10.1002/hbm.24552. 30784139 10.1002/hbm.24552 PMC6508987

[97] Passarotti AM, Sweeney JA, Pavuluri MN. Neural correlates of response inhibition in pediatric bipolar disorder and attention deficit hyperactivity disorder (vol 181, pg 36, 2010). Psychiat Res-Neuroim. 2014;222:172. 10.1016/j.pscychresns.2014.03.007. 10.1016/j.pscychresns.2009.07.002 PMC2795009 19926457

[98] Rubia K, Halari R, Mohammad AM, Taylor E, Brammer M. Methylphenidate normalizes frontocingulate underactivation during error processing in attention-deficit/hyperactivity disorder. Biol Psychiatry. 2011;70:255–62. 10.1016/j.biopsych.2011.04.018. 21664605 10.1016/j.biopsych.2011.04.018 PMC3139835

[99] Rubia K, Overmeyer S, Taylor E, Brammer M, Williams SC, Simmons A, et al. Hypofrontality in attention deficit hyperactivity disorder during higher-order motor control: a study with functional MRI. Am J Psychiat. 1999;156:891–6. 10360128 10.1176/ajp.156.6.891

[100] Rubia K, Smith AB, Brammer MJ, Toone B, Taylor E. Abnormal brain activation during inhibition and error detection in medication-naive adolescents with ADHD. Am J psychiatry. 2005;162:1067–75. 10.1176/appi.ajp.162.6.1067. 15930054 10.1176/appi.ajp.162.6.1067

[101] Siniatchkin M, Glatthaar N, von Müller GG, Prehn-Kristensen A, Wolff S, Knöchel S, et al. Behavioural treatment increases activity in the cognitive neuronal networks in children with attention deficit/hyperactivity disorder. Brain Topography. 2012;25:332–44. 10.1007/s10548-012-0221-6. 22392009 10.1007/s10548-012-0221-6

[102] Stevens MC, Pearlson GD, Calhoun VD, Bessette KL. Functional neuroimaging evidence for distinct neurobiological pathways in attention-deficit/hyperactivity disorder. Biol Psychiat-Cogn N. 2018;3:675–85. 10.1016/j.bpsc.2017.09.005. 10.1016/j.bpsc.2017.09.005 PMC6089229 30092917

[103] Suskauer SJ, Simmonds DJ, Caffo BS, Denckla MB, Pekar JJ, Mostofsky SH. fMRI of intrasubject variability in ADHD: anomalous premotor activity with prefrontal compensation. J Am Acad Child Adolesc Psychiatry. 2008;47:1141–50. 10.1097/CHI.0b013e3181825b1f. 18724253 10.1097/CHI.0b013e3181825b1f PMC3932630

[104] Tamm L, Menon V, Ringel J, Reiss AL. Event-related fMRI evidence of frontotemporal involvement in aberrant response inhibition and task switching in attention-deficit/hyperactivity disorder. J Am Acad Child Adolesc Psychiatry. 2004;43:1430–40. 10.1097/01.chi.0000140452.51205.8d. 10.1097/01.chi.0000140452.51205.8d 15502603

[105] Thornton S, Bray S, Langevin LM, Dewey D. Functional brain correlates of motor response inhibition in children with developmental coordination disorder and attention deficit/hyperactivity disorder. Hum Mov Sci. 2018;59:134–42. 10.1016/j.humov.2018.03.018. 29655169 10.1016/j.humov.2018.03.018

[106] van Rooij D, Hoekstra PJ, Mennes M, von Rhein D, Thissen AJ, Heslenfeld D, et al. Distinguishing Adolescents With ADHD from their unaffected siblings and healthy comparison subjects by neural activation patterns during response inhibition. Am J Psychiat. 2015;172:674–83. 10.1176/appi.ajp.2014.13121635. 25615565 10.1176/appi.ajp.2014.13121635 PMC4490085

[107] Cai W, Griffiths K, Korgaonkar MS, Williams LM, Menon V. Inhibition-related modulation of salience and fronto-parietal networks predicts cognitive control ability and inattention in children with ADHD. Mol Psychiatr. 2021;26:4016–25. 10.1038/s41380-019-0564-4. 10.1038/s41380-019-0564-4 PMC7188596 31664176

[108] Gao Z, Duberg K, Warren SL, Zheng L, Hinshaw SP, Menon V, et al. Reduced temporal and spatial stability of neural activity patterns predict cognitive control deficits in children with ADHD. Nat Commun. 2025;16:2346. 10.1038/s41467-025-57685-x. 40057478 10.1038/s41467-025-57685-x PMC11890578

[109] Nugiel T, Roe MA, Engelhardt LE, Mitchell ME, Zheng A, Church JA. Pediatric ADHD symptom burden relates to distinct neural activity across executive function domains. Neuroimage-Clin. 2020;28:102394. 10.1016/j.nicl.2020.102394. 32971467 10.1016/j.nicl.2020.102394 PMC7511724

[110] Iannaccone R, Hauser TU, Ball J, Brandeis D, Walitza S, Brem S. Classifying adolescent attention-deficit/hyperactivity disorder (ADHD) based on functional and structural imaging. Eur Child Adoles Psy. 2015;24:1279–89. 10.1007/s00787-015-0678-4. 10.1007/s00787-015-0678-4 25613588

[111] Cortese S, Kelly C, Chabernaud C, Proal E, Di Martino A, Milham MP, et al. Toward systems neuroscience of ADHD: a meta-analysis of 55 fMRI studies. Am J psychiatry. 2012;169:1038–55. 10.1176/appi.ajp.2012.11101521. 22983386 10.1176/appi.ajp.2012.11101521 PMC3879048

[112] van Rooij D, Hartman CA, Mennes M, Oosterlaan J, Franke B, Rommelse N, et al. Altered neural connectivity during response inhibition in adolescents with attention-deficit/hyperactivity disorder and their unaffected siblings. NeuroImage Clin. 2015;7:325–35. 10.1016/j.nicl.2015.01.004. 25610797 10.1016/j.nicl.2015.01.004 PMC4297885

[113] McLaren DG, Ries ML, Xu G, Johnson SC. A generalized form of context-dependent psychophysiological interactions (gPPI): a comparison to standard approaches. NeuroImage. 2012;61:1277–86. 10.1016/j.neuroimage.2012.03.068. 22484411 10.1016/j.neuroimage.2012.03.068 PMC3376181

[114] Geurts HM, Verte S, Oosterlaan J, Roeyers H, Sergeant JA. How specific are executive functioning deficits in attention deficit hyperactivity disorder and autism?. J child Psychol psychiatry, allied Discip. 2004;45:836–54. 10.1111/j.1469-7610.2004.00276.x. 10.1111/j.1469-7610.2004.00276.x 15056314

[115] Purvis KL, Tannock R. Phonological processing, not inhibitory control, differentiates ADHD and reading disability. J Am Acad Child Adolesc Psychiatry. 2000;39:485–94. 10.1097/00004583-200004000-00018. 10761351 10.1097/00004583-200004000-00018

[116] Sergeant JA. Modeling attention-deficit/hyperactivity disorder: a critical appraisal of the cognitive-energetic model. Biol Psychiatry. 2005;57:1248–55. 10.1016/j.biopsych.2004.09.010. 15949995 10.1016/j.biopsych.2004.09.010

[117] Sonuga-Barke EJ. The dual pathway model of AD/HD: an elaboration of neuro-developmental characteristics. Neurosci Biobehav Rev. 2003;27:593–604. 14624804 10.1016/j.neubiorev.2003.08.005

[118] Tripp G, Wickens JR. Research review: dopamine transfer deficit: a neurobiological theory of altered reinforcement mechanisms in ADHD. J child Psychol psychiatry, allied Discip. 2008;49:691–704. 10.1111/j.1469-7610.2007.01851.x. 10.1111/j.1469-7610.2007.01851.x 18081766

[119] Luman M, Tripp G, Scheres A. Identifying the neurobiology of altered reinforcement sensitivity in ADHD: A review and research agenda. Neurosci Biobehav Rev. 2010;34:744–54. 10.1016/j.neubiorev.2009.11.021. 19944715 10.1016/j.neubiorev.2009.11.021

[120] Tripp G, Wickens JR. Neurobiology of ADHD. Neuropharmacology. 2009;57:579–89. 10.1016/j.neuropharm.2009.07.026. 19627998 10.1016/j.neuropharm.2009.07.026

[121] Carlson CL, Booth JE, Shin MS, Canu WH. Parent-, teacher-, and self-rated motivational styles in ADHD subtypes. J Learn Disabil-Us. 2002;35:104–13. 10.1177/002221940203500202. 10.1177/002221940203500202 15490739

[122] Colomer C, Berenguer C, Rosello B, Baixauli I, Miranda A. The impact of inattention, hyperactivity/impulsivity symptoms, and executive functions on learning behaviors of children with ADHD. Front Psychol. 2017;8:540. 10.3389/fpsyg.2017.00540. 28446885 10.3389/fpsyg.2017.00540 PMC5388736

[123] Gut J, Heckmann C, Meyer CS, Schmid M, Grob A. Language skills, mathematical thinking, and achievement motivation in children with ADHD, disruptive behavior disorders, and normal controls. Learn Individ Differ. 2012;22:375–9. 10.1016/j.lindif.2011.12.002.

[124] Smith ZR, Langberg JM. Review of the evidence for motivation deficits in youth with ADHD and their association with functional outcomes. Clin Child Fam Psych. 2018;21:500–26. 10.1007/s10567-018-0268-3. 10.1007/s10567-018-0268-3 30141121

[125] Morsink S, Sonuga-Barke E, Van der Oord S, Van Dessel J, Lemiere J, Danckaerts M. Task-related motivation and academic achievement in children and adolescents with ADHD. Eur Child Adoles Psy. 2021;30:131–41. 10.1007/s00787-020-01494-8. 10.1007/s00787-020-01494-8 32157390

[126] Hoza B, Pelham WE, Waschbusch DA, Kipp H, Owens JS. Academic task persistence of normally achieving ADHD and control boys: Performance, self-evaluations, and attributions. J consulting Clin Psychol. 2001;69:271–83. 10.1037//0022-006x.69.2.271 11393604

[127] Ogg J, Volpe R, Rogers M. Understanding the relationship between inattention and early literacy trajectories in kindergarten. Sch Psychol Quart. 2016;31:565–82. 10.1037/spq0000130. 10.1037/spq0000130 26551254

[128] DuPaul GJ, Langberg JM. In Attention-deficit/hyperactivity disorder: A handbook for diagnosis and treatment (4th edn) (ed RA Barkely) (Guilford., 2014).

[129] Addicott MA, Schechter JC, Sapyta JJ, Selig JP, Kollins SH, Weiss MD. Methylphenidate increases willingness to perform effort in adults with ADHD. Pharmacol Biochem Be. 2019;183:14–21. 10.1016/j.pbb.2019.06.008. 10.1016/j.pbb.2019.06.008 PMC6628703 31226260

[130] Chong TTJ, Fortunato E, Bellgrove MA. Amphetamines improve the motivation to invest effort in attention-deficit/hyperactivity disorder. J Neurosci. 2023;43:6898–908. 10.1523/Jneurosci.0982-23.2023. 37666665 10.1523/JNEUROSCI.0982-23.2023 PMC10573750

[131] Winter Y, Ben-Pazi H, Pollak Y. Effort Allocation in Children With ADHD: abnormal decision-making or poor execution?. J Atten Disord. 2019;23:1240–50. 10.1177/1087054716654569. 27329487 10.1177/1087054716654569

[132] Mies GW, Ma I, de Water E, Buitelaar JK, Scheres A. Waiting and working for rewards: attention-deficit/hyperactivity disorder is associated with steeper delay discounting linked to amygdala activation, but not with steeper effort discounting. Cortex. 2018;106:164–73. 10.1016/j.cortex.2018.05.018. 30005368 10.1016/j.cortex.2018.05.018

[133] Bardach L, Murayama K. The role of rewards in motivation-Beyond dichotomies. Learn Instr. 2025;96:102056. 10.1016/j.learninstruc.2024.102056.

[134] Daniel TL, Esser JK. Intrinsic Motivation as Influenced by Rewards, Task Interest, and Task Structure. J Appl Psychol. 1980;65:566–73. 10.1037/0021-9010.65.5.566.

[135] Deci EL, Koestner R, Ryan RM. A meta-analytic review of experiments examining the effects of extrinsic rewards on intrinsic motivation. Psychological Bull. 1999;125:627–68. 10.1037/0033-2909.125.6.627. 10.1037/0033-2909.125.6.627 10589297

[136] Blain B, Pinhorn I, Sharot T. Sensitivity to intrinsic rewards is domain general and related to mental health. Nat Ment Health. 2023;1:679–91. 10.1038/s44220-023-00116-x. 38665692 10.1038/s44220-023-00116-x PMC11041740

[137] Antrop I, Stock P, Verté S, Wiersema JR, Baeyens D, Roeyers H. ADHD and delay aversion:: the influence of non-temporal stimulation on choice for delayed rewards. J Child Psychol Psyc. 2006;47:1152–8. 10.1111/j.1469-7610.2006.01619.x. 10.1111/j.1469-7610.2006.01619.x 17076754

[138] Kuntsi J, Oosterlaan J, Stevenson J. Psychological mechanisms in hyperactivity: I response inhibition deficit, working memory impairment, delay aversion, or something else?. J Child Psychol Psyc. 2001;42:199–210. 10.1111/1469-7610.00711. 11280416

[139] Solanto MV, Abikoff H, Sonuga-Barke E, Schachar R, Logan GD, Wigal T, et al. The ecological validity of delay aversion and response inhibition as measures of impulsivity in AD/HD: a supplement to the NIMH multimodal treatment study of AD/HD. J Abnorm child Psychol. 2001;29:215–28. 10.1023/a:1010329714819. 11411784 10.1023/a:1010329714819

[140] Jackson JN, MacKillop J. Attention-Deficit/hyperactivity disorder and monetary delay discounting: a meta-analysis of case-control studies. Biol Psychiatry Cogn Neurosci Neuroimaging. 2016;1:316–25. 10.1016/j.bpsc.2016.01.007. 27722208 10.1016/j.bpsc.2016.01.007 PMC5049699

[141] Patros CH, Alderson RM, Kasper LJ, Tarle SJ, Lea SE, Hudec KL. Choice-impulsivity in children and adolescents with attention-deficit/hyperactivity disorder (ADHD): A meta-analytic review. Clin Psychol Rev. 2016;43:162–74. 10.1016/j.cpr.2015.11.001. 26602954 10.1016/j.cpr.2015.11.001

[142] Abler B, Herrnberger B, Gron G, Spitzer M. From uncertainty to reward: BOLD characteristics differentiate signaling pathways. BMC Neurosci. 2009;10:154. 10.1186/1471-2202-10-154. 20028546 10.1186/1471-2202-10-154 PMC2806405

[143] Grabenhorst F, Rolls ET. Value, pleasure and choice in the ventral prefrontal cortex. Trends Cognit Sci. 2011;15:56–67. 10.1016/j.tics.2010.12.004. 21216655 10.1016/j.tics.2010.12.004

[144] Haber SN, Knutson B. The reward circuit: linking primate anatomy and human imaging. Neuropsychopharmacology. 2010;35:4–26. 10.1038/npp.2009.129. 19812543 10.1038/npp.2009.129 PMC3055449

[145] Knutson B, Adams CM, Fong GW, Hommer D. Anticipation of increasing monetary reward selectively recruits nucleus accumbens. J Neurosci. 2001;21:RC159. 11459880 10.1523/JNEUROSCI.21-16-j0002.2001 PMC6763187

[146] Morelli SA, Sacchet MD, Zaki J. Common and distinct neural correlates of personal and vicarious reward: A quantitative meta-analysis. NeuroImage. 2015;112:244–53. 10.1016/j.neuroimage.2014.12.056. 25554428 10.1016/j.neuroimage.2014.12.056 PMC4408229

[147] Sescousse G, Caldú X, Segura B, Dreher JC. Processing of primary and secondary rewards: a quantitative meta-analysis and review of human functional neuroimaging studies. Neurosci Biobehav Rev. 2013;37:681–96. 10.1016/j.neubiorev.2013.02.002. 23415703 10.1016/j.neubiorev.2013.02.002

[148] Knutson B, Greer SM. Anticipatory affect: neural correlates and consequences for choice. Philos T R Soc B. 2008;363:3771–86. 10.1098/rstb.2008.0155. 10.1098/rstb.2008.0155 PMC2607363 18829428

[149] Oldham S, Murawski C, Fornito A, Youssef G, Yücel M, Lorenzetti V. The anticipation and outcome phases of reward and loss processing: A neuroimaging meta-analysis of the monetary incentive delay task. Hum brain Mapp. 2018;39:3398–418. 10.1002/hbm.24184. 29696725 10.1002/hbm.24184 PMC6055646

[150] Cao Z, Bennett M, Orr C, Icke I, Banaschewski T, Barker GJ, et al. Mapping adolescent reward anticipation, receipt, and prediction error during the monetary incentive delay task. Hum brain Mapp. 2019;40:262–83. 10.1002/hbm.24370. 30240509 10.1002/hbm.24370 PMC6865381

[151] Carmona S, Hoekzema E, Ramos-Quiroga JA, Richarte V, Canals C, Bosch R, et al. Response inhibition and reward anticipation in medication-naive adults with attention-deficit/hyperactivity disorder: a within-subject case-control neuroimaging study. Hum brain Mapp. 2012;33:2350–61. 10.1002/hbm.21368. 21826761 10.1002/hbm.21368 PMC6870239

[152] Edel MA, Enzi B, Witthaus H, Tegenthoff M, Peters S, Juckel G, et al. Differential reward processing in subtypes of adult attention deficit hyperactivity disorder. J Psychiatr Res. 2013;47:350–6. 10.1016/j.jpsychires.2012.09.026. 23201229 10.1016/j.jpsychires.2012.09.026

[153] Furukawa E, Bado P, da Costa R, Melo B, Erthal P, de Oliveira IP, et al. Reward modality modulates striatal responses to reward anticipation in ADHD: Effects of affiliative and food stimuli. Psychiat Res-Neuroim. 2022;327:111561. 10.1016/j.pscychresns.2022.111561. 10.1016/j.pscychresns.2022.111561 36334392

[154] Furukawa E, Bado P, Tripp G, Mattos P, Wickens JR, Bramati IE, et al. Abnormal Striatal BOLD Responses to Reward Anticipation and Reward Delivery in ADHD. PLoS one. 2014;9:e89129. 10.1371/journal.pone.0089129. 24586543 10.1371/journal.pone.0089129 PMC3935853

[155] Hoogman M, Aarts E, Zwiers M, Slaats-Willemse D, Naber M, Onnink M, et al. Nitric oxide synthase genotype modulation of impulsivity and ventral striatal activity in adult ADHD patients and healthy comparison subjects. Am J Psychiat. 2011;168:1099–106. 10.1176/appi.ajp.2011.10101446. 21724667 10.1176/appi.ajp.2011.10101446

[156] Paloyelis Y, Mehta MA, Faraone SV, Asherson P, Kuntsi J. Striatal sensitivity during reward processing in attention-deficit/hyperactivity disorder. J Am Acad Child Adolesc Psychiatry. 2012;51:722–32. 10.1016/j.jaac.2012.05.006. 22721595 10.1016/j.jaac.2012.05.006 PMC3763946

[157] Scheres A, Milham MP, Knutson B, Castellanos FX. Ventral striatal hyporesponsiveness during reward anticipation in attention-deficit/hyperactivity disorder. Biol Psychiatry. 2007;61:720–4. 10.1016/j.biopsych.2006.04.042. 16950228 10.1016/j.biopsych.2006.04.042

[158] Stoy M, Schlagenhauf F, Schlochtermeier L, Wrase J, Knutson B, Lehmkuhl U, et al. Reward processing in male adults with childhood ADHD-a comparison between drug-naive and methylphenidate-treated subjects. Psychopharmacology. 2011;215:467–81. 10.1007/s00213-011-2166-y. 21298512 10.1007/s00213-011-2166-y

[159] Ströhle A, Stoy M, Wrase J, Schwarzer S, Schlagenhauf F, Huss M, et al. Reward anticipation and outcomes in adult males with attention-deficit/hyperactivity disorder. NeuroImage. 2008;39:966–72. 10.1016/j.neuroimage.2007.09.044. 17996464 10.1016/j.neuroimage.2007.09.044

[160] van Hulst BM, de Zeeuw P, Bos DJ, Rijks Y, Neggers SF, Durston S. Children with ADHD symptoms show decreased activity in ventral striatum during the anticipation of reward, irrespective of ADHD diagnosis. J Child Psychol Psyc. 2017;58:206–14. 10.1111/jcpp.12643. 10.1111/jcpp.12643 27678006

[161] von Rhein D, Cools R, Zwiers MP, van der Schaaf M, Franke B, Luman M, et al. Increased neural responses to reward in adolescents and young adults with attention-deficit/hyperactivity disorder and their unaffected siblings. J Am Acad Child Adolesc Psychiatry. 2015;54:394–402. 10.1016/j.jaac.2015.02.012. 25901776 10.1016/j.jaac.2015.02.012 PMC4417499

[162] Plichta MM, Vasic N, Wolf RC, Lesch KP, Brummer D, Jacob C, et al. Neural hyporesponsiveness and hyperresponsiveness during immediate and delayed reward processing in adult attention-deficit/hyperactivity disorder. Biol Psychiat. 2009;65:7–14. 10.1016/j.biopsych.2008.07.008. 18718573 10.1016/j.biopsych.2008.07.008

[163] Braver TS, Krug MK, Chiew KS, Kool W, Westbrook JA, Clement NJ, et al. Mechanisms of motivation-cognition interaction: challenges and opportunities. Cogn Affect Behav Ne. 2014;14:443–72. 10.3758/s13415-014-0300-0. 10.3758/s13415-014-0300-0 PMC4986920 24920442

[164] Yee DM, Braver TS. Interactions of motivation and cognitive control. Curr Opin Behav Sci. 2018;19:83–90. 10.1016/j.cobeha.2017.11.009. 30035206 10.1016/j.cobeha.2017.11.009 PMC6051692

[165] Boehler CN, Hopf JM, Stoppel CM, Krebs RM. Motivating inhibition - reward prospect speeds up response cancellation. Cognition. 2012;125:498–503. 10.1016/j.cognition.2012.07.018. 22921189 10.1016/j.cognition.2012.07.018

[166] Leotti LA, Wager TD. Motivational influences on response inhibition measures. J Exp psychology Hum Percept Perform. 2010;36:430–47. 10.1037/a0016802. 10.1037/a0016802 PMC3983778 20364928

[167] Cascone AD, Calabro F, Foran W, Larsen B, Nugiel T, Parr AC, et al. Brain tissue iron neurophysiology and its relationship with the cognitive effects of dopaminergic modulation in children with and without ADHD. Developmental Cognit Neurosci. 2023;63:101274. 10.1016/j.dcn.2023.101274. 10.1016/j.dcn.2023.101274 PMC10372187 37453207

[168] Dekkers TJ, Agelink van Rentergem JA, Koole A, van den Wildenberg W, Popma A, Bexkens A, et al. Time-on-task effects in children with and without ADHD: depletion of executive resources or depletion of motivation?. Eur Child Adoles Psy. 2017;26:1471–81. 10.1007/s00787-017-1006-y. 10.1007/s00787-017-1006-y PMC5701950 28536846

[169] Demurie E, Roeyers H, Wiersema JR, Sonuga-Barke E. No evidence for inhibitory deficits or altered reward processing in ADHD: data from a new integrated monetary incentive delay go/no-go task. J Atten Disord. 2016;20:353–67. 10.1177/1087054712473179. 23382578 10.1177/1087054712473179

[170] Desman C, Petermann F, Hampel P. Deficit in response inhibition in children with attention deficit/hyperactivity disorder (ADHD): impact of motivation?. Child Neuropsychol. 2008;14:483–503. 10.1080/09297040701625831. 18982507 10.1080/09297040701625831

[171] Fosco WD, Hawk L, Rosch KS, Bubnik MG. Evaluating cognitive and motivational accounts of greater reinforcement effects among children with attention-deficit/hyperactivity disorder. Behav Brain Funct. 2015;11:20. 10.1186/s12993-015-0065-9. 25926127 10.1186/s12993-015-0065-9 PMC4438621

[172] Gomez R. Underlying processes in the poor response inhibition of children with Attention-Deficit/Hyperactivity Disorder. J Atten Disord. 2003;6:111–22. 10.1177/108705470300600303. 12821876 10.1177/108705470300600303

[173] Groom MJ, Scerif G, Liddle PF, Batty MJ, Liddle EB, Roberts KL, et al. Effects of motivation and medication on electrophysiological markers of response inhibition in children with attention-deficit/hyperactivity disorder. Biol Psychiat. 2010;67:624–31. 10.1016/j.biopsych.2009.09.029. 19914599 10.1016/j.biopsych.2009.09.029 PMC2845810

[174] Huang-Pollock CL, Mikami AY, Pfiffner L, McBurnett K. ADHD subtype differences in motivational responsivity but not inhibitory control: evidence from a reward-based variation of the stop signal paradigm. J Clin child Adolesc Psychol. 2007;36:127–36. 10.1080/15374410701274124. 17484686 10.1080/15374410701274124

[175] Iaboni F, Douglas VI, Baker AG. Effects of Reward and Response Costs on Inhibition in Adhd Children. J Abnorm Psychol. 1995;104:232–40. 10.1037/0021-843x.104.1.232. 7897047 10.1037/0021-843X.104.1.232

[176] Kohls G, Herpertz-Dahlmann B, Konrad K. Hyperresponsiveness to social rewards in children and adolescents with attention-deficit/hyperactivity disorder (ADHD). Behav Brain Funct. 2009;5:20. 10.1186/1744-9081-5-20. 19426488 10.1186/1744-9081-5-20 PMC2685404

[177] Konrad K, Gauggel S, Manz A, Scholl M. Lack of inhibition: a motivational deficit in children with attention deficit/hyperactivity disorder and children with traumatic brain injury. Child Neuropsychol. 2000;6:286–96. 10.1076/chin.6.4.286.3145. 11992192 10.1076/chin.6.4.286.3145

[178] Liddle EB, Hollis C, Batty MJ, Groom MJ, Totman JJ, Liotti M, et al. Task-related default mode network modulation and inhibitory control in ADHD: effects of motivation and methylphenidate. J Child Psychol Psyc. 2011;52:761–71. 10.1111/j.1469-7610.2010.02333.x. 10.1111/j.1469-7610.2010.02333.x PMC4754961 21073458

[179] Michel JA, Kerns KA, Mateer CA. The effect of reinforcement variables on inhibition in children with ADHD. Child Neuropsychol. 2005;11:295–302. 10.1080/092970490911270. 16036453 10.1080/092970490911270

[180] Miyasaka M, Nomura M. Effect of financial and non-financial reward and punishment for inhibitory control in boys with attention deficit hyperactivity disorder. Res developmental disabilities. 2023;134:104438. 10.1016/j.ridd.2023.104438. 10.1016/j.ridd.2023.104438 36701956

[181] Oosterlaan J, Logan GD, Sergeant JA. Response inhibition in AD/HD, CD, comorbid AD/HD + CD, anxious, and control children: a meta-analysis of studies with the stop task. J child Psychol psychiatry, allied Discip. 1998;39:411–25. 9670096

[182] Rosch KS, Fosco WD, Pelham WE Jr, Waxmonsky JG, Bubnik MG, Hawk LW Jr. Reinforcement and stimulant medication ameliorate deficient response inhibition in children with attention-deficit/hyperactivity disorder. J Abnorm child Psychol. 2016;44:309–21. 10.1007/s10802-015-0031-x. 25985978 10.1007/s10802-015-0031-x PMC4654720

[183] Shanahan MA, Pennington BR, Willcutt EW. Do motivational incentives reduce the inhibition deficit in ADHD?. Developmental neuropsychology. 2008;33:137–59. 10.1080/87565640701884238. 18443974 10.1080/87565640701884238

[184] Sinopoli KJ, Schachar R, Dennis M. Traumatic brain injury and secondary attention-deficit/hyperactivity disorder in children and adolescents: The effect of reward on inhibitory control. J Clin Exp Neuropsyc. 2011;33:805–19. 10.1080/13803395.2011.562864. 10.1080/13803395.2011.562864 PMC3184364 21598155

[185] Slusarek M, Velling S, Bunk D, Eggers C. Motivational effects on inhibitory control in children with ADHD. J Am Acad Child Adolesc Psychiatry. 2001;40:355–63. 10.1097/00004583-200103000-00016. 11288778 10.1097/00004583-200103000-00016

[186] Stevens J, Quittner AL, Zuckerman JB, Moore S. Behavioral inhibition, self-regulation of motivation, and working memory in children with attention deficit hyperactivity disorder. Developmental neuropsychol. 2002;21:117–39. 10.1207/S15326942dn2102_1. 10.1207/S15326942DN2102_1 12139195

[187] Uebel H, Albrecht B, Asherson P, Börger NA, Butler L, Chen W, et al. Performance variability, impulsivity errors and the impact of incentives as gender-independent endophenotypes for ADHD. J Child Psychol Psyc. 2010;51:210–8. 10.1111/j.1469-7610.2009.02139.x. 10.1111/j.1469-7610.2009.02139.x PMC2921046 19929943

[188] Van der Meere J, Marzocchi GM, De Meo T. Response inhibition and attention deficit hyperactivity disorder with and without oppositional defiant disorder screened from a community sample. Developmental neuropsychol. 2005;28:459–72. 10.1207/s15326942dn2801_1. 10.1207/s15326942dn2801_1 15992251

[189] Wodka EL, Mahone EM, Blankner JG, Larson JC, Fotedar S, Denckla MB, et al. Evidence that response inhibition is a primary deficit in ADHD. J Clin Exp Neuropsyc. 2007;29:345–56. 10.1080/13803390600678046. 10.1080/13803390600678046 17497558

[190] Scheres A, Oosterlaan J, Sergeant JA. Response inhibition in children with DSM-IV subtypes of AD/HD and related disruptive disorders: the role of reward. Child Neuropsychol. 2001;7:172–89. 10.1076/chin.7.3.172.8746. 12187474 10.1076/chin.7.3.172.8746

[191] Boehler CN, Schevernels H, Hopf JM, Stoppel CM, Krebs RM. Reward prospect rapidly speeds up response inhibition via reactive control. Cognitive, Affect Behav Neurosci. 2014;14:593–609. 10.3758/s13415-014-0251-5. 10.3758/s13415-014-0251-5 24448735

[192] Le TM, Zhang S, Zhornitsky S, Wang WY, Li CSR. Neural correlates of reward-directed action and inhibition of action. Cortex. 2020;123:42–56. 10.1016/j.cortex.2019.10.007. 31747630 10.1016/j.cortex.2019.10.007

[193] Lee HJ, Lin FH, Kuo WJ. The neural mechanism underpinning balance calibration between action inhibition and activation initiated by reward motivation. Sci Rep-Uk. 2017;7:9722. 10.1038/s41598-017-10539-z. 10.1038/s41598-017-10539-z PMC5575270 28852156

[194] Qiao L, Xu L, Che X, Zhang L, Li Y, Xue G, et al. The Motivation-Based Promotion of Proactive Control: The Role of Salience Network. Front Hum Neurosci. 2018;12:328. 10.3389/fnhum.2018.00328. 30154707 10.3389/fnhum.2018.00328 PMC6103265

[195] Lee B, Cai W, Young CB, Yuan R, Ryman S, Kim J, et al. Latent brain state dynamics and cognitive flexibility in older adults. Prog Neurobiol. 2022;208:102180. 10.1016/j.pneurobio.2021.102180. 34627994 10.1016/j.pneurobio.2021.102180 PMC9585912

[196] Epstein JN, Langberg JM, Rosen PJ, Graham A, Narad ME, Antonini TN, et al. Evidence for Higher Reaction Time Variability for Children With ADHD on a range of cognitive tasks including reward and event rate manipulations. Neuropsychology. 2011;25:427–41. 10.1037/a0022155. 21463041 10.1037/a0022155 PMC3522094

[197] Fassbender C, Zhang H, Buzy WM, Cortes CR, Mizuiri D, Beckett L, et al. A lack of default network suppression is linked to increased distractibility in ADHD. Brain Res. 2009;1273:114–28. 10.1016/j.brainres.2009.02.070. 19281801 10.1016/j.brainres.2009.02.070 PMC4721585

[198] Sidlauskaite J, Sonuga-Barke E, Roeyers H, Wiersema JR. Default mode network abnormalities during state switching in attention deficit hyperactivity disorder. Psychological Med. 2016;46:519–28. 10.1017/S0033291715002019. 10.1017/S0033291715002019 26456561

[199] Metin B, Krebs RM, Wiersema JR, Verguts T, Gasthuys R, van der Meere JJ, et al. Dysfunctional modulation of default mode network activity in attention-deficit/hyperactivity disorder. J Abnorm Psychol. 2015;124:208–14. 10.1037/abn0000013. 25314265 10.1037/abn0000013

[200] Hutchison RM, Womelsdorf T, Allen EA, Bandettini PA, Calhoun VD, Corbetta M, et al. Dynamic functional connectivity: promise, issues, and interpretations. NeuroImage. 2013;80:360–78. 10.1016/j.neuroimage.2013.05.079. 23707587 10.1016/j.neuroimage.2013.05.079 PMC3807588

[201] Misra J, Pessoa L. Brain dynamics and spatiotemporal trajectories during threat processing. eLife. 10.7554/eLife.102539.3. 10.7554/eLife.102539 PMC12854673 41609638

[202] Ryali S, Supekar K, Chen T, Kochalka J, Cai W, Nicholas J, et al. Temporal dynamics and developmental maturation of salience, default and central-executive network interactions revealed by variational bayes hidden markov modeling. PLoS computational Biol. 2016;12:e1005138. 10.1371/journal.pcbi.1005138. 10.1371/journal.pcbi.1005138 PMC5154470 27959921

[203] Song H, Shim WM, Rosenberg MD. Large-scale neural dynamics in a shared low-dimensional state space reflect cognitive and attentional dynamics. Elife. 2023;12:e85487. 10.7554/eLife.85487. 37395724 10.7554/eLife.85487 PMC10400080

[204] Yamashita A, Rothlein D, Kucyi A, Valera EM, Esterman M. Brain state-based detection of attentional fluctuations and their modulation. NeuroImage. 2021;236:118072. 10.1016/j.neuroimage.2021.118072. 33882346 10.1016/j.neuroimage.2021.118072

[205] Vyas S, Golub MD, Sussillo D, Shenoy KV. Computation Through Neural Population Dynamics. Annu Rev Neurosci. 2020;43:249–75. 10.1146/annurev-neuro-092619-4115. 32640928 10.1146/annurev-neuro-092619-094115 PMC7402639

[206] Prince JS, Charest I, Kurzawski JW, Pyles JA, Tarr MJ, Kay KN. Improving the accuracy of single-trial fMRI response estimates using GLMsingle. Elife. 2022;11:e77599. 10.7554/eLife.77599. 36444984 10.7554/eLife.77599 PMC9708069

[207] Zeithamova D, Sanchez MAD, Adke A. Trial timing and pattern-information analyses of fMRI data. NeuroImage. 2017;153:221–31. 10.1016/j.neuroimage.2017.04.025. 28411155 10.1016/j.neuroimage.2017.04.025

[208] Kohoutová L, Atlas LY, Büchel C, Buhle JT, Geuter S, Jepma M, et al. Individual variability in brain representations of pain. Nat Neurosci. 2022;25:749–759. 10.1038/s41593-022-01081-x. 35637368 10.1038/s41593-022-01081-x PMC9435464

[209] Koolschijn RS, Emir UE, Pantelides AC, Nili H, Behrens T, Barron HC. The Hippocampus and Neocortical Inhibitory Engrams Protect against Memory Interference. Neuron. 2019;101:528–541.e6. 10.1016/j.neuron.2018.11.042. 30581011 10.1016/j.neuron.2018.11.042 PMC6560047

[210] Miller JA, Tambini A, Kiyonaga A, D’Esposito M. Long-term learning transforms prefrontal cortex representations during working memory. Neuron. 2022;110:3805–3819. 10.1016/j.neuron.2022.09.019. 36240768 10.1016/j.neuron.2022.09.019 PMC9768795

[211] Park SA, Miller DS, Boorman ED. Inferences on a multidimensional social hierarchy use a grid-like code. Nat Neurosci. 2021;24:1292–301. 10.1038/s41593-021-00916-3. 34465915 10.1038/s41593-021-00916-3 PMC8759596

[212] Faraone SV, Biederman J, Spencer TJ, Aleardi M. Comparing the efficacy of medications for ADHD using meta-analysis. MedGenMed. 2006;8:4. 17415287 PMC1868385

[213] Faraone SV, Buitelaar J. Comparing the efficacy of stimulants for ADHD in children and adolescents using meta-analysis. Eur Child Adoles Psy. 2010;19:353–64. 10.1007/s00787-009-0054-3. 10.1007/s00787-009-0054-3 19763664

[214] Boland H, DiSalvo M, Fried R, Woodworth KY, Wilens T, Faraone SV, et al. A literature review and meta-analysis on the effects of ADHD medications on functional outcomes. J Psychiatr Res. 2020;123:21–30. 10.1016/j.jpsychires.2020.01.006. 32014701 10.1016/j.jpsychires.2020.01.006

[215] Van der Oord S, Prins PJM, Oosterlaan J, Emmelkamp PMG. Efficacy of methylphenidate, psychosocial treatments and their combination in school-aged children with ADHD: A meta-analysis. Clin Psychol Rev. 2008;28:783–800. 10.1016/j.cpr.2007.10.007. 18068284 10.1016/j.cpr.2007.10.007

[216] Wilens TE. Effects of methylphenidate on the catecholaminergic system in attention-deficit/hyperactivity disorder. J Clin Psychopharm. 2008;28:S46–S53. 10.1097/JCP.0b013e318173312f. 10.1097/JCP.0b013e318173312f 18480677

[217] Coghill DR, Seth S, Pedroso S, Usala T, Currie J, Gagliano A. Effects of methylphenidate on cognitive functions in children and adolescents with attention-deficit/hyperactivity disorder: evidence from a systematic review and a meta-analysis. Biol Psychiat. 2014;76:603–15. 10.1016/j.biopsych.2013.10.005. 24231201 10.1016/j.biopsych.2013.10.005

[218] Isfandnia F, El Masri S, Radua J, Rubia K. The effects of chronic administration of stimulant and non-stimulant medications on executive functions in ADHD: a systematic review and meta-analysis. Neurosci Biobehav Rev. 2024;162:105703. 10.1016/j.neubiorev.2024.105703. 38718988 10.1016/j.neubiorev.2024.105703

[219] Tamminga HGH, Reneman L, Huizenga HM, Geurts HM. Effects of methylphenidate on executive functioning in attention-deficit/hyperactivity disorder across the lifespan: a meta-regression analysis. Psychological Med. 2016;46:1791–807. 10.1017/S0033291716000350. 10.1017/S0033291716000350 27019103

[220] Mizuno Y, Cai W, Supekar K, Makita K, Takiguchi S, Silk TJ, et al. Methylphenidate enhances spontaneous fluctuations in reward and cognitive control networks in children with attention-deficit/hyperactivity disorder. Biol Psychiatry Cogn Neurosci Neuroimaging. 2023;8:271–80. 10.1016/j.bpsc.2022.10.001. 36717325 10.1016/j.bpsc.2022.10.001 PMC12994527

[221] Mizuno Y, Cai W, Supekar K, Makita K, Takiguchi S, Tomoda A, et al. Methylphenidate remediates aberrant brain network dynamics in children with attention-deficit/hyperactivity disorder: a randomized controlled trial. NeuroImage. 2022;257:119332. 10.1016/j.neuroimage.2022.119332. 35640787 10.1016/j.neuroimage.2022.119332 PMC9286726

[222] Chelonis JJ, Johnson TA, Ferguson SA, Berry KJ, Kubacak B, Edwards MC, et al. Effect of methylphenidate on motivation in children with attention-deficit/hyperactivity disorder. Exp Clin Psychopharm. 2011;19:145–53. 10.1037/a0022794. 10.1037/a0022794 21463072

[223] Shiels K, Hawk LW, Reynolds B, Mazzullo RJ, Rhodes JD, Pelham WE, et al. Effects of methylphenidate on discounting of delayed rewards in attention deficit/hyperactivity disorder. Exp Clin Psychopharm. 2009;17:291–301. 10.1037/a0017259. 10.1037/a0017259 PMC2908283 19803628

[224] Westbrook A, Braver TS. Dopamine does double duty in motivating cognitive effort. Neuron. 2016;91:708. 10.1016/j.neuron.2016.07.020. 27497225 10.1016/j.neuron.2016.07.020

[225] Manza P, Tomasi D, Shokri-Kojori E, Zhang R, Kroll D, Feldman D, et al. Neural circuit selective for fast but not slow dopamine increases in drug reward. Nat Commun. 2023;14:6408. 10.1038/s41467-023-41972-6. 37938560 10.1038/s41467-023-41972-6 PMC10632365

[226] Tomasi D, Manza P, Yan W, Shokri-Kojori E, Demiral ŞB, Yonga MV, et al. Examining the role of dopamine in methylphenidate’s effects on resting brain function. Proc Natl Acad Sci USA. 2023;120:e2314596120. 10.1073/pnas.2314596120. 38109535 10.1073/pnas.2314596120 PMC10756194

[227] Cubillo A, Smith AB, Barrett N, Giampietro V, Brammer MJ, Simmons A, et al. Shared and drug-specific effects of atomoxetine and methylphenidate on inhibitory brain dysfunction in medication-naive ADHD Boys. Cereb cortex. 2014;24:174–85. 10.1093/cercor/bhs296. 23048018 10.1093/cercor/bhs296 PMC3862268

[228] Rubia K, Halari R, Cubillo A, Mohammad AM, Brammer M, Taylor E. Methylphenidate normalises activation and functional connectivity deficits in attention and motivation networks in medication-naive children with ADHD during a rewarded continuous performance task. Neuropharmacology. 2009;57:640–52. 10.1016/j.neuropharm.2009.08.013. 19715709 10.1016/j.neuropharm.2009.08.013

[229] Rubia K, Alegria AA, Cubillo AI, Smith AB, Brammer MJ, Radua J. Effects of stimulants on brain function in attention-deficit/hyperactivity disorder: a systematic review and meta-analysis. Biol Psychiat. 2014;76:616–28. 10.1016/j.biopsych.2013.10.016. 24314347 10.1016/j.biopsych.2013.10.016 PMC4183380

[230] Berridge CW, Devilbiss DM. Psychostimulants as cognitive enhancers: the prefrontal cortex, catecholamines, and attention-deficit/hyperactivity disorder. Biol Psychiat. 2011;69:E101–E111. 10.1016/j.biopsych.2010.06.023. 20875636 10.1016/j.biopsych.2010.06.023 PMC3012746

[231] Volkow ND, Wang G, Fowler JS, Logan J, Gerasimov M, Maynard L, et al. Therapeutic doses of oral methylphenidate significantly increase extracellular dopamine in the human brain. J Neurosci. 2001;21:U1–U5. 10.1523/JNEUROSCI.21-02-j0001.2001 PMC6763805 11160455

[232] Arnsten AFT. The emerging neurobiology of attention deficit hyperactivity disorder: the key role of the prefrontal association. Cortex J Pediatr-Us. 2009;154:S22–S31. 10.1016/j.jpeds.2009.01.018. 10.1016/j.jpeds.2009.01.018 PMC2894421 20596295

[233] Faraone SV. The pharmacology of amphetamine and methylphenidate: Relevance to the neurobiology of attention-deficit/hyperactivity disorder and other psychiatric comorbidities. Neurosci Biobehav Rev. 2018;87:255–70. 10.1016/j.neubiorev.2018.02.001. 29428394 10.1016/j.neubiorev.2018.02.001 PMC8063758

[234] Heal DJ, Smith SL, Gosden J, Nutt DJ. Amphetamine, past and present - a pharmacological and clinical perspective. J Psychopharmacol. 2013;27:479–96. 10.1177/0269881113482532. 23539642 10.1177/0269881113482532 PMC3666194

[235] Schultz W. Multiple dopamine functions at different time courses. Annu Rev Neurosci. 2007;30:259–88. 10.1146/annurev.neuro.28.061604.135722. 17600522 10.1146/annurev.neuro.28.061604.135722

[236] Bymaster FP, Katner JS, Nelson DL, Hemrick-Luecke SK, Threlkeld PG, Heiligenstein JH, et al. Atomoxetine increases extracellular levels of norepinephrine and dopamine in prefrontal cortex of rat: a potential mechanism for efficacy in attention deficit/hyperactivity disorder. Neuropsychopharmacology. 2002;27:699–711. 12431845 10.1016/S0893-133X(02)00346-9

[237] Chamberlain SR, Del Campo N, Dowson J, Müller U, Clark L, Robbins TW, et al. Atomoxetine improved response inhibition in adults with attention deficit/hyperactivity disorder. Biol Psychiat. 2007;62:977–84. 10.1016/j.biopsych.2007.03.003. 17644072 10.1016/j.biopsych.2007.03.003

[238] Griffiths KR, Leikauf JE, Tsang TW, Clarke S, Hermens DF, Efron D, et al. Response inhibition and emotional cognition improved by atomoxetine in children and adolescents with ADHD: the ACTION randomized controlled trial. J Psychiatr Res. 2018;102:57–64. 10.1016/j.jpsychires.2018.03.009. 29674270 10.1016/j.jpsychires.2018.03.009 PMC9148271

[239] Arnsten AF, Scahill L, Findling RL. alpha2-Adrenergic receptor agonists for the treatment of attention-deficit/hyperactivity disorder: emerging concepts from new data. J Child Adolesc Psychopharmacol. 2007;17:393–406. 10.1089/cap.2006.0098. 17822336 10.1089/cap.2006.0098

[240] Arnsten AFT. Guanfacine’s mechanism of action in treating prefrontal cortical disorders: Successful translation across species. Neurobiol Learn Mem. 2020;176:107327. 10.1016/j.nlm.2020.107327. 33075480 10.1016/j.nlm.2020.107327 PMC7567669

[241] Wang M, Ramos BP, Paspalas CD, Shu Y, Simen A, Duque A, et al. α2A-adrenoceptors strengthen working memory networks by inhibiting cAMP-HCN channel signaling in prefrontal cortex. Cell. 2007;129:397–410. 10.1016/j.cell.2007.03.015. 17448997 10.1016/j.cell.2007.03.015

[242] Cai WD, Mizuno Y, Tomoda A, Menon V. Bayesian dynamical system analysis of the effects of methylphenidate in children with attention-deficit/hyperactivity disorder: a randomized trial. Neuropsychopharmacology. 2023;48:1690–8. 10.1038/s41386-023-01668-3. 37491674 10.1038/s41386-023-01668-3 PMC10516959

[243] Pessiglione M, Vinckier F, Bouret S, Daunizeau J, Le Bouc R. Why not try harder? Computational approach to motivation deficits in neuro-psychiatric diseases. Brain. 2018;141:629–50. 10.1093/brain/awx278. 29194534 10.1093/brain/awx278

[244] Yee DM. Neural and computational mechanisms of motivation and decision-making. J Cognit Neurosci. 2024;36:2822–30. 10.1162/jocn_a_02258. 39378176 10.1162/jocn_a_02258 PMC11602011

[245] Eshel N, Touponse GC, Wang AR, Osterman AK, Shank AN, Groome AM, et al. Striatal dopamine integrates cost, benefit, and motivation. Neuron. 2024;112:500–514.e5. 10.1016/j.neuron.2023.10.038. 38016471 10.1016/j.neuron.2023.10.038 PMC10922131

